# Second-Generation Linkage Maps for the Pacific Oyster *Crassostrea gigas* Reveal Errors in Assembly of Genome Scaffolds

**DOI:** 10.1534/g3.115.019570

**Published:** 2015-08-06

**Authors:** Dennis Hedgecock, Grace Shin, Andrew Y. Gracey, David Van Den Berg, Manoj P. Samanta

**Affiliations:** * Department of Biological Sciences, University of Southern California, Los Angeles, California 90089-0371; † Preventive Medicine, Keck School of Medicine, University of Southern California, Los Angeles, California 90089-9601; ‡ Systemix Institute, Redmond, Washington 98053

**Keywords:** Illumina GoldenGate bead assays, regression maps, maximum likelihood maps, distortion of segregation ratios, genotyping error

## Abstract

The Pacific oyster *Crassostrea gigas*, a widely cultivated marine bivalve mollusc, is becoming a genetically and genomically enabled model for highly fecund marine metazoans with complex life-histories. A genome sequence is available for the Pacific oyster, as are first-generation, low-density, linkage and gene-centromere maps mostly constructed from microsatellite DNA markers. Here, higher density, second-generation, linkage maps are constructed from more than 1100 coding (exonic) single-nucleotide polymorphisms (SNPs), as well as 66 previously mapped microsatellite DNA markers, all typed in five families of Pacific oysters (nearly 172,000 genotypes). The map comprises 10 linkage groups, as expected, has an average total length of 588 cM, an average marker-spacing of 1.0 cM, and covers 86% of a genome estimated to be 616 cM. All but seven of the mapped SNPs map to 618 genome scaffolds; 260 scaffolds contain two or more mapped SNPs, but for 100 of these scaffolds (38.5%), the contained SNPs map to different linkage groups, suggesting widespread errors in scaffold assemblies. The 100 misassembled scaffolds are significantly longer than those that map to a single linkage group. On the genetic maps, marker orders and intermarker distances vary across families and mapping methods, owing to an abundance of markers segregating from only one parent, to widespread distortions of segregation ratios caused by early mortality, as previously observed for oysters, and to genotyping errors. Maps made from framework markers provide stronger support for marker orders and reasonable map lengths and are used to produce a consensus high-density linkage map containing 656 markers.

The Pacific oyster *Crassostrea gigas* is one of the most widely cultivated marine species, having been introduced for this purpose from Asia to all continents but Antarctica (Mann 1979; Food and Agriculture Organization 2015). Global annual production is uncertain, owing to taxonomic confusion in reports from China and other countries (Wang *et al.* 2008), but is conservatively estimated as 555,914 metric tons in 2013 (Food and Agriculture Organization 2015), down from 4.6 million metric tons of cupped oysters grouped under the Pacific oyster moniker in 2006. Because of its commercial importance, a number of breeding programs have been initiated over the years (Hershberger *et al.* 1984; Langdon *et al.* 2003; Hedgecock and Davis 2007; Dégremont *et al.* 2010), substantial genetic and genomic resources have been developed [genetic lines; expressed sequence tag (EST) collections; transcriptomes; bacterial artificial chromosome libraries; Hedgecock *et al.* 2005; Curole and Hedgecock 2007], and recently the genome has been sequenced (Zhang *et al.* 2012). The Pacific oyster is also becoming a model species for research on environmental resilience (Applebaum *et al.* 2014).

Linkage maps are essential tools useful for mapping quantitative-trait loci (QTL; Lander and Botstein 1989; Doerge 2002), marker-assisted selection, positional cloning, and genome assembly. Yet, construction of a single, high-density consensus linkage map for a species, particularly for one such as the Pacific oyster, which has a high load of early lethal or highly deleterious mutations (Launey and Hedgecock 2001; Plough and Hedgecock 2011), is a challenging goal.

Previously published linkage maps for the Pacific oyster were based on microsatellite DNA markers (Hubert and Hedgecock 2004; Hubert *et al.* 2009; Plough and Hedgecock 2011), on dominant amplified fragment-length polymorphism (AFLP) markers or combinations of AFLP and microsatellite markers (Li and Guo 2004; Guo *et al.* 2012), and more recently (Sauvage *et al.* 2010; Zhong *et al.* 2014) on microsatellite markers together with single-nucleotide polymorphisms (SNPs). Although adequate for mapping viability QTL (Plough and Hedgecock 2011; Plough 2012), these first-generation maps have rather low marker densities. Here, higher density, second-generation linkage maps are constructed for the Pacific oyster, based largely on assays of a fixed set of 1536 coding (exonic), SNPs in five mapping families.

Variability in marker orders and intermarker distances among individual family maps and between different mapping methods presents formidable challenges to the construction of a consensus linkage map for this species. Harmonizing results from regression (RG) and maximum likelihood (ML) mapping methods, we identify three factors behind uncertainties in marker orders and inflated map distances: (i) markers segregating from only one parent, (ii) markers linked to chromosomal regions impacted by strong, early viability selection, and (iii) genotyping errors. In the end, a single consensus map, constructed from the most reliable set of individual framework maps, is achieved. SNP linkage information has an unexpected bearing on the current genome assembly, insofar as it implies that a large proportion of genome scaffolds may be incorrectly assembled.

## Materials and Methods

### Mapping families

Five full-sib families of Pacific oysters were used for linkage mapping. Two were F_2_ families, 2 × 10 (n = 90) and 51 × 35 (n = 108), each derived from an intercross of full-sib F_1_ hybrids, which, in turn, were produced by crosses of partially inbred lines (*i.e.*, 2, 10; 51, 35; Hedgecock and Davis 2007). Three other families, F12, F20, and F45 (each, n = 46), were produced by crosses between adult oysters taken in 2006 from the wild population in Dabob Bay, Washington (L. V. Plough, G. Shin, and D. Hedgecock, unpublished data); these random-bred, full-sib families were the G_0_ foundation stock for development of inbred lines (Hedgecock and Davis 2007).

### EST library sequencing and transcriptome assembly

To construct libraries of expressed DNA sequences, RNA was extracted from 2-yr-old oysters, from G_3_ inbred lines 51 and 35, after we subjected small groups to different stressful conditions. The pedigree of all oysters was confirmed by typing microsatellite DNA markers on the inbred oysters and tissue samples from their parents (Curole and Hedgecock 2007). RNA was pooled within the inbred lines and made into coded cDNA libraries. Four additional larval cDNA libraries, two from 2-hr to 12-hr-old larvae and two from 15-hr to 30-hr-old larvae, were also constructed.

These libraries were Sanger-sequenced by the U.S. DOE Joint Genome Institute (JGI; larval libraries CBSI, CBSN, CBSO, and CBSP; and adult libraries CCBN, CCTP, and CCTS; GenBank accessions HS109651-HS248985). The adult libraries contributed 126,331 (90.7%) of the 139,335 EST sequences obtained from these libraries. Additional sequence was generated from a putative, nonredundant subset of 11,904 cDNA clones, which were pooled, their inserts fragmented,and sequenced by Illumina Genome Analyzer methods (SRA accession SRR2119182). Further sequence information came from two other larval cDNA libraries, CFPP and CFPS, which were made, respectively, from 6-d- and 18-d-old larvae of a reciprocal hybrid between inbred lines 35 and 51; these libraries were sequenced by 454 GS FLX Titanium methods (GenBank accessions SRX032364 and SRX032365). Sequences from all libraries were assembled, using miraEST (Chevreux *et al.* 2004), into a *C. gigas* transcriptome comprising 52,095 unigenes.

### SNP discovery, marker development, and genotyping

The JGI ESTs (145,600 sequences) were compared with the transcriptome assembly, using BLAST (blastn, cutoff 1e-50) and the best hit for each EST was aligned with the EST sequence using CLUSTAL. Alignments of cDNA sequences from the two inbred lines 51 and 35 were combined to generate a SAM file for all ESTs. Separately, reads from two additional Illumina RNA-Seq libraries (SRP061799) were compared with the assembled transcriptome, using BWA (Li and Durbin 2009; http://bio-bwa.sourceforge.net/). The three SAM output files were combined, using the pileup command in SAMtools (Li *et al.* 2009; http://www.htslib.org/) to find 126,392 sites with potential variants (whether true SNPs or read errors). Candidate SNPs were sorted according to the amount of supporting sequence evidence and whether they were segregating within or between the two inbred lines; each alternative allele had to be supported by two or more sequences. Additionally, the unigene contigs were mapped on a draft version of the oyster genome, so that intron-exon boundaries could be identified. The list of high-quality SNPs was further refined to remove candidates located within 50 nucleotides of an intron-exon boundary, so that a 100-nt GoldenGate assay probe centered on the SNP site (Shen *et al.* 2005) would be contained entirely within a single exon. A total of 4122, 100-nt probes were selected, after these steps, for design of GoldenGate probes.

From the list of potential probes, we chose the top 1536 SNPs for the final GoldenGate bead array (Shen *et al.* 2005), which was used to genotype four 96-well plates containing the five mapping families. Subsequently, 27 SNPs were found to have duplicates in the array, 6 SNPs were present in triplicate, and 1 SNP was present in quadruplicate; these repeats were removed from the mapping data sets and the subsequent tally of SNPs and genome scaffolds mapped. Although present in earlier assemblies of the genome, which enabled identification of exon boundaries, seven of the assayed SNPs were not present in the final genome assembly; these retain the “USC” reference numbers from the transcriptome assembly. Otherwise, SNPs were assigned names based on the genome scaffold number and nucleotide position (see *Genomic locations and annotations of mapped, coding SNPs*).

To determine genotyping error in the GoldenGate bead assay, nine individual samples were run in duplicate on plates processed at the same time. Discounting for missing data and duplicated markers, an average of 1437 SNPs were compared in duplicate samples, a total of 12,937 genotypes; no discrepancies between duplicated genotypes were found.

In addition to SNPs assayed by the GoldenGate assay, we genotyped 66 microsatellite DNA markers that have been mapped in previous studies (Hubert and Hedgecock 2004, Hubert *et al.* 2009; Plough and Hedgecock 2011, Plough 2012). We also typed 17 SNPs in families F45 and 51 × 35, which were identified early by the SNP discovery pipeline but genotyped on a Sequenome platform (L. V. Plough, G. Shin, and D. Hedgecock, unpublished data). Across families and markers, a total of 179,263 genotypes were determined in this study and contributed to the linkage maps to be described. Complete genotype data sets for the five families are given in Supporting Information, Table S1, Table S2, Table S3, Table S4, and Table S5.

### Linkage analyses

Linkage mapping calculations were made with JoinMap 4.1 (Van Ooijen 2006), using the cross-pollinated coding of genotypes to accommodate the multiple mating types observed in the full-sib families (Table 1; here, but not in Table S1, Table S2, Table S3, Table S4, and Table S5, coding of markers segregating from only one parent is reversed from JoinMap convention, to reflect our long-standing practice of writing controlled crosses as ♂×♀). Markers missing data for more than 10% of individuals in a family were excluded. Initial groupings of markers were based on independence log of the odds (LOD) tests in steps from a LOD of 1.0 (independence *P* threshold, 0.001; recombination threshold, 0.25) to a LOD of 10.0 (independence *P* threshold, 0.0001; recombination threshold, 0.05). The independence LOD is less likely than the linkage LOD test to find spurious linkage, when segregation distortion, such as that observed for oysters (Bierne *et al.* 1998; Launey and Hedgecock 2001; Plough and Hedgecock 2011), is present (Van Ooijen 2006). The final grouping LOD is the maximum threshold value passed by all markers in at least one test of independence with other markers in the group. Previously mapped microsatellite markers (Hubert and Hedgecock 2004; Plough and Hedgecock 2011) were used to associate the resulting groupings with the 10 identified linkage groups (LG). In each family, sets of markers with identical genotypes were identified in subsequent mapping steps. Since identical markers map to the same location, we used only one marker from each set of identical markers, to increase the efficiency of linkage calculations, and we subsequently positioned initially excluded identical markers at the locus where their representative mapped.

**Table 1:** Distribution of markers by mating types (♂×♀) in five mapping families of the Pacific oyster *Crassostrea gigas*

Mating Type	12	45	20	2 × 10	51 × 35	Sum
*np × nn*	219	232	223	122	61	857
*ll × lm*	221	246	240	146	67	920
*hk × hk*	134	134	132	236	396	1032
*ef × eg*	13	10	8	15	8	54
*ab × cd*	15	28	13	2	0	58
All	602	650	616	521	532	2921

As observed previously for the Pacific oyster (Launey and Hedgecock 2001; Plough and Hedgecock 2011; Plough 2012), we find widespread distortions of Mendelian segregation ratios in most linkage groups, as reflected by highly significant χ^2^ goodness-of-fit tests. The extent of such distortions on maps is summarized by the proportions of markers on a LG that show departures from expected Mendelian segregation ratios at a nominal significance threshold of *α* ≤ 0.01. A two-way analysis of variance on arcsine square root transformations of these proportions is used to assess variance among families and linkage groups.

Both RG (Stam 1993) and ML (Jansen *et al.* 2001) mapping methods, as implemented in JoinMap 4.1, were used to construct linkage maps. Initial RG linkage maps were made with all markers, using default settings (maximum recombination frequency of 0.4, minimum LOD of 1.0, goodness-of-fit jump threshold for removing loci of 5.0), Kosambi’s mapping function, three-rounds of fitting (a second round attempts to add loci removed in the first round, with a third round forcing all markers onto the map), and a ripple performed after each marker addition. Kosambi’s mapping function is justified by evidence for crossover interference (Hubert *et al.* 2009). Occasionally, insufficient linkage or failure to determine linkage phase with default settings required relaxation of thresholds. In three cases (LG 2 in 51 × 35 and LG 8 and LG 10 in F20, the latter two requiring relaxation of linkage thresholds), single markers that appeared to cause poor fit or excessive map lengths were removed before proceeding with mapping. Nearest-neighbor fit—the sum of the absolute values of the differences between observed pairwise and calculated map distances (in centiMorgans, cM) of each marker to its nearest informative neighbors on either side—is used as an indicator of the quality of the map order.

The ML mapping method uses multipoint, ML-based algorithms to determine optimal intermarker distances and marker order and simulated annealing to provide statistical support for marker order. Distance in the ML method is calculated with the Haldane mapping function, which assumes no cross-over interference and may overestimate distances when interference is present. We used the JoinMap default spatial sampling thresholds (0.10, 0.05, 0.03, 0.02, and 0.01) and three rounds of optimization per sample. Default parameters for map-order optimization were used except that chain length was increased from 10,000 to 20,000. Parameters for multipoint estimation of recombination frequency were generally increased (length of burn-in chain, from 10,000 to 20,000; number of Monte Carlo EM cycles, from 4 to 10; chain length per EM cycles, from 1000 to 5000) to ensure convergence.

We attempted to resolve discrepancies in map orders and marker distances between RG and ML maps by focusing, first, on biparentally segregating framework markers and then on a set of well-spaced anchor loci. When suitable congruence between RG and ML maps for anchor loci was achieved, we applied the resulting order as a fixed order to the mapping of framework markers and all markers. Maps of framework markers were used to construct consensus maps and to compare pairwise recombination rates between male and female parents.

The length of the genome was estimated for each family by two methods. First, we calculated *s*, the average spacing of markers, as the sum of the lengths of the 10 linkage maps within each family divided by the total number of mapped intervals (*i.e.*, the total number of markers minus the number of linkage groups, 10). Genome length was then estimated as the sum of linkage group lengths plus 20*s*, to account for intervals beyond the most distal markers on both ends of the 10 linkage groups (following Fishman *et al.* 2001). We also estimated genome length for each family as the sum of map lengths multiplied by (*m* + 1)/(*m* − 1), where *m* is the number of markers per linkage group (Chakravarti *et al.* 1991). Finally, for each estimate of map length, we estimated genome coverage as 1- *e*^-2^*^sn^*^/^*^L^*, where *s* is average spacing, *n* is total number of markers assigned to linkage groups, and *L* is the total map length (Bishop *et al.* 1983).

A consensus map was made, using MergeMap (Wu *et al.* 2011); owing to the explicit statistical support for marker order that the ML method provides, we merged family ML framework maps. Prior to merging, we removed poor-fitting markers from some family maps to improve agreement in map lengths and marker-orders between RG and ML maps. The G_0_ families did not have enough bi-parentally segregating markers to provide framework maps for LG 9. In addition, we removed LG 2 and LG 7 maps for G_0_ families *F20* and *F45* from the final consensus map, because they fit a preliminary merged map poorly; likewise, we removed *F12* and *F20* framework maps from the final consensus map for LG 10. Families were weighted in MergeMap, roughly by the number of individuals typed, such that the G_0_ full-sib families were weighted 1.0 and the two F_2_ families were weighted 2.0. The inverse Haldane mapping function was used to convert ML map units to recombination fractions, which were then converted into Kosambi map units prior to submitting to MergeMap. The consensus maps produced by MergeMap still appeared to have inflated distances, compared to the individual maps, so, after adjusting individual map origins to the appropriate consensus-map position, we performed linear regressions of individual maps onto consensus maps in order to adjust final distances on each consensus linkage map.

### Genomic locations and annotations of mapped, coding SNPs

The SNP-containing sequences were aligned with the reference genome using BWA-MEM (H. Li; http://arxiv.org/abs/1303.3997) and each SNP location was identified using a custom script. Loci were named (*e.g.*, 3-G98282A) according to the genome scaffold to which they aligned (3 in the example), followed by the nucleotide position within that scaffold (98,282 in the example); the position is preceded by the base in the reference genome (G) and followed by the substitution observed in that strand (A).

### Data availability

Accession numbers to sequences used for SNP discovery are given in “EST library sequencing and transcriptome assembly” and in “SNP discovery, marker development and genotyping.” Supporting information at http://www.g3journal.org/content/suppl/2015/08/04/g3.115.019570.DC1 contains detailed descriptions and links to Excel Table S1, Table S2, Table S3, Table S4, Table S5, Table S6, and Table S7, containing the genotype-input files for JoinMap, the mapping of SNPs to scaffolds and to linkage groups, and the consensus linkage map. Tables 6 and 7 provide a roadmap to parameters used in sequential steps of the linkage mapping analyses.

## Results

### Polymorphism of markers and preliminary assignment to LGs

Each of the mapped 1172 SNPs or microsatellite DNA markers can be classified into one of five possible parental mating types (♂×♀), *np* × *nn*, *ll* × *lm*, *hk* × *hk*, *ef* × *eg*, and *ab* × *cd*, depending on whether parent-pairs have two, three, or four alleles. The distribution of markers across these categories (Table 1) reflects the types of markers assayed and levels of marker polymorphism in the populations from which the parents were drawn. There is a preponderance of two-allele types (96.2%), as expected for the bulk of bi-allelic SNP markers. Across the three G_0_ families, proportions of two-allele mating types are homogeneous (0.38 for *np* × *nn*, 0.40 for *ll* × *lm*, and 0.22 for *hk* × *hk*; χ^2^ = 0.65, 4 df, *P* = 0.96), but the two F_2_ families have many more *hk* × *hk* mating types, reflecting derivation from a cross of inbred grandparents (*i.e.*, *hh* × *kk*). The two F_2_ families differ significantly from each other, however, with 51 × 35 having a much greater relative proportion of *hk* × *hk* mating types than *2 × 10* (χ^2^ = 89.8, 2 df, *P* < 0.0001); this difference is consistent with the initial selection of SNPs fixed for different alleles in the 51 *vs.* 35 inbred parent lines. Microsatellite DNA markers account for all of the *ab* × *cd* mating types and most of the *ef* × *eg* mating types. Some SNPs fall into the *ef* × *eg* category, owing to inference of a shared null allele (*i.e.*, apparent *aa* × *cc* mating types produce progeny types, *aa*, *cc*, *ac*, and blanks; such crosses were re-interpreted as *aØ* × *cØ*, where *Ø* is a non-amplifying null allele, yielding *aØ*, *cØ*, *ac*, and *ØØ* progeny). In keeping with their inbred history, the two F_2_ families differ noticeably from the three, random-bred G_0_ families, in having almost 100 fewer markers mapped (526 *vs.* 623) and many fewer *ab* × *cd* mating types (1.0 *vs.* 18.7, on average; Table 1).

Markers are initially grouped through pairwise *G*-tests of independent segregation. Summing across all five mapping families, a total 1085 coding SNPs are grouped, together with enough previously mapped microsatellite DNA markers, 66 in total, to establish consistency with previous linkage maps (Hubert and Hedgecock 2004; Hubert *et al.* 2009; Plough and Hedgecock 2011). Markers are provisionally mapped, using the regression mapping method, yielding 10 linkage groups in each family, as expected, with total lengths ranging from 508 to 690 cM and an average total map length of 588 cM (Table 2). With a mean of 584 markers per map, including identical loci, these maps have an average marker spacing of 1.04 cM. The 50 linkage groups range in size from 28.7 to 198.4 cM, with an average of 59 cM. Only five linkage groups (one each for LGs 1, 3, 4, 7, and 8) are greater than 85 cM in length, and these appear to be outliers with respect to map lengths obtained in the other families (Table 2); we return to these excessively long linkage groups in a later section. Genome lengths are estimated for each family by two methods (Fishman *et al.* 2001; Chakravarti *et al.* 1991) and range from 524 to 744 cM, with averages of 609 and 623 cM, respectively (Table 2). Despite variability in genome lengths among families, genome coverages are 85% or 86% in all cases (Table 2).

**Table 2:** Lengths and numbers of markers for initial regression maps, by linkage group, for five mapping families of the Pacific oyster *Crassostrea gigas*

Linkage Group	Length, No. Markers	Families					
		12	45	20	2 × 10	51 × 35	Averages*^a^*
1	Length, cM	43.3	56.7	65.4	96.4	57.4	63.8
	No. markers	80	81	94	76	86	83.4
2	Length, cM	37.1	41.0	28.7	42.4	51.7	41.8
	No. markers	9	13	10	4	12	9.6
3	Length, cM	53.1	48.8	45.4	100.0	51.6	63.4
	No. markers	77	75	70	56	58	67.2
4	Length, cM	57.0	73.4	95.0	71.8	60.8	65.7
	No. markers	57	77	58	40	53	57.0
5	Length, cM	49.3	49.2	55.4	53.1	61.9	58.7
	No. markers	35	37	41	31	28	34.4
6	Length, cM	47.0	68.4	42.8	65.4	58.0	56.3
	No. markers	96	98	89	78	71	86.4
7	Length, cM	54.0	44.8	30.0	94.8	67.0	58.1
	No. markers	85	88	90	75	81	83.8
8	Length, cM	198.4	33.4	78.1	68.8	48.1	85.3
	No. markers	41	53	45	35	47	44.2
9	Length, cM	38.8	39.0	42.1	35.1	57.8	42.6
	No. markers	15	16	14	21	19	17.0
10	Length, cM	50.8	52.9	52.2	62.0	65.7	56.1
	No. markers	107	112	105	105	77	101.2
	Sum of lengths	628.8	507.6	535.0	689.8	579.9	588.2
	Total no. of markers*^b^*	602	650	616	521	532	584.2
	Average spacing, cM*^c^*	1.06	0.79	0.88	1.35	1.11	1.04
	Genome length 1*^d^*	650.1	523.5	552.7	716.8	602.1	609.0
	Genome length 2*^e^*	664.2	531.7	562.9	744.5	612.9	623.3
	Genome coverage 1*^f^*	0.86	0.86	0.86	0.86	0.86	0.86
	Genome coverage 2*^g^*	0.85	0.86	0.86	0.85	0.85	0.85

a Averages for Length and no. markers are arithmetic.

b Includes previously mapped microsatellite DNA markers (Hubert and Hedgecock 2004; Plough and Hedgecock 2011).

c Calculated as the total length of all linkage groups divided by the total number of map intervals, which is the total number of markers minus the number of linkage groups.

d Calculated following Fishman *et al.* (2001).

e Calculated following Chakravarti *et al.* (1991).

f Calculated following Bishop *et al.* (1983), using genome length 1.

g Calculated following Bishop *et al.* (1983), using genome length 2.

Most SNPs in the GoldenGate assay (823 of 1085, 75.9%) are mapped in two or more families (Table 3), lending confidence to LG assignments; of these, only two (1255-G326792C and 43940-T130339C) map to different linkage groups in different families. Close inspection of the mapping support for these markers did not uncover any explanation, as it did in a dozen similar cases for which data transcription errors were discovered and corrected. These two markers are dropped from further linkage analyses.

**Table 3:** Distribution of SNP markers over five mapping families of the Pacific oyster *Crassostrea gigas*

No. of Maps	No. of SNPs
1	262
2	285
3	290
4	180
5	68
Sum	1085

SNP, single-nucleotide polymorphism.

### Mapping of genome scaffolds

**Figure 1:**
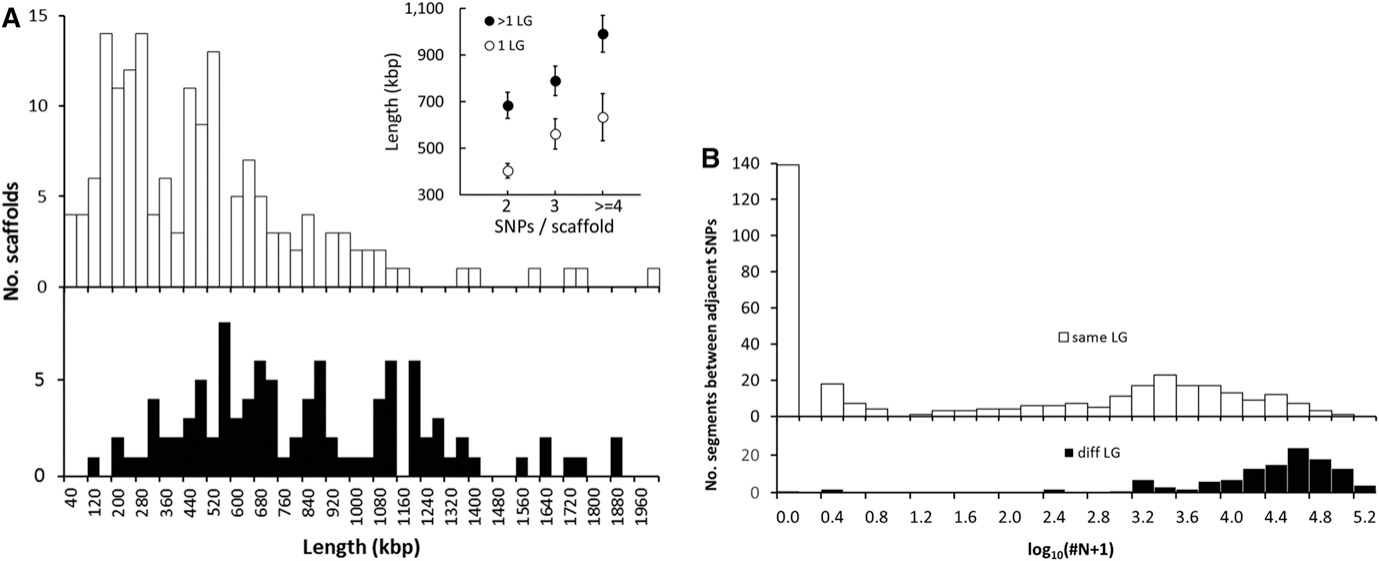
Comparisons of scaffolds or adjacent single-nucleotide polymorphisms (SNPs) on a scaffold that either do (unfilled symbols or bars) or do not (filled symbols or bars) map to one linkage group. (A) Distribution of the lengths of scaffolds in these two categories; inset shows average lengths of scaffolds classified by number of SNPs per scaffold. (B) Distributions of the number of ambiguous bases (N in the genome assembly) between adjacent SNPs that either do or do not map to the same linkage group. The number of Ns is transformed to log_10_(#N+1).

All but seven of the 1085 mapped SNPs can be located on genome scaffolds (Zhang *et al.* 2012; Table S6). Of the 618 scaffolds with mapped SNPs, 358 contain one SNP, and 260 contain two or more SNPs (Table 4). Of the scaffolds with two or more SNPs, 100 (38.5%) contain SNPs that unexpectedly map to two or more linkage groups (Table 5). Cross-classifying scaffolds by the number of SNPs they contain and by the number of linkage groups to which they map reveals a highly significant, positive association between the two factors (contingency χ^2^ = 31.84, 3 d.f., for four levels of SNPs per scaffold—2, 3, 4, and >4 SNPs—and two classes of mapping—to one linkage group or to more than one linkage group; *P* < < 0.0001). The median length of the 100 scaffolds that map to two or more linkage groups, 740 kbp, is significantly longer than the median length of the 160 scaffolds that map to a single linkage group, 403 kbp (Figure 1A), and the more SNPs per scaffold, the longer the average multiply-mapped scaffold compared to the average singly-mapped scaffold (Figure 1A inset). Average distance between adjacent SNPs that map to different linkage groups is 273 kbp (*n* = 118), whereas the average distance between adjacent SNPs that map to the same linkage group is 58.2 kbp (*n* = 337; *t* = 11.14, *P* < < 0.0001, two-tailed test of difference in sample means with unequal variances). Numbers of ambiguous bases (coded as N in the published genome), between adjacent SNPs on a scaffold that either do or do not map to the same linkage group, are also significantly different (Figure 1B). The distribution of the number of Ns between adjacent SNPs mapping to the same linkage group has a mode at zero and a median of 11, whereas between adjacent SNPs that map to different linkage groups, the distribution has a mode at nearly 40,000 and a median just over 25,000 ambiguous bases.

**Table 4:** Numbers of mapped, coding SNPs in genome scaffolds of the Pacific oyster *Crassostrea gigas*

No. of SNPs per scaffold	No. of Scaffolds	Total No. of SNPs
1	358	358
2	143	286
3	69	207
4	26	104
5	13	65
6	5	30
7	4	28
All	618	1078
≥2	260	720

SNP, single-nucleotide polymorphism.

**Table 5:** Number of scaffolds with two or more SNPs that map to one or more than one linkage group, by number of SNPs per scaffold

No. of SNPs per scaffold	Number of Linkage Groups to Which Scaffolds Map				Sum
	1	2	3	4	
2	106	37	0	0	143
3	39	27	3	0	69
4	11	13	2	0	26
5	3	4	5	1	13
6	0	3	2	0	5
7	1	0	3	0	4
Sum	160	84	15	1	260

SNP, single-nucleotide polymorphism.

Finally, we consider the number of “breakpoints” necessary to account for the order with which same-scaffold SNPs are mapped to linkage groups. For example, a scaffold with three SNPs mapping to two linkage groups (*A* and *B*) can have one breakpoint (LG-assignment orders, *AAB* or *ABB*) or two breakpoints (*ABA* or *BAB*); of the 27 scaffolds in this category (Table 5), 26 have the minimum of one breakpoint and only one has two breakpoints. Similarly, of the 13 scaffolds with four SNPs mapping to two linkage groups, all 13 have one breakpoint (6, *AAAB* and 7, *AABB*) and none has an order requiring two breakpoints (*AABA*) or three breakpoints (*ABAB*). Indeed, across the 60 informative combinations in Table 5 (*i.e.*, cases in which the number of SNPs > number of LG assignments), only two cases require more than the minimum number of breakpoints.

### Comparison of RG and ML maps

Provisional linkage maps made by the RG method generally provide poor fits to observed recombination rates among markers (Table 6, nonidentical loci only). Threshold LOD values for grouping range from 3 to 10, reflecting differences in the number of progeny genotyped, such that the two larger F_2_ families have average LOD values near 9 and the three smaller G_0_ families have average LOD values near 5. In seven of 50 cases (five families × 10 linkage groups), default thresholds for linkage had to be relaxed to permit all markers to be added. For 44 of 50 maps, linkage of all markers could only be achieved by forcing remaining markers onto a third-round RG map, sometimes resulting in large contributions to total χ^2^ or negative distances to existing mapped markers; on average, 10.6 makers were forced onto the 44 linkage groups, with a range from one to 42 markers. Maximum values for the nearest-neighbor fit statistic range from a low of 0.2 cM, for LG 2 in 2 × 10, to 621.3 cM, for LG 10 in the same family; indeed, 36 of 50 fit statistics exceed the estimated lengths of their linkage groups (Table 6). Maximum nearest neighbor fit is significantly greater for forced than for unforced maps (252.6 cM *vs.* 74.0 cM, *F_1,48_* = 4.612, *P* = 0.037).

**Table 6:** Summary statistics for regression and maximum likelihood maps for all markers grouped in 10 LG, in five families of the Pacific oyster

LG	Family	No. Markers	Grouping LOD*^a^*	No. Markers Used	RG Type*^b^*	Map round	No. markers forced	RG length, cM	RG Max. NNFit	Prop. Markers Distorted	ML Type*^c^*	ML Length, cM	ML Max. NNFit	RG *v*. ML *r^2^*
1	51 × 35	68	10	66	RGFO	3	4	57.4	507.7	0.000	MLFO	222.0	8.8	0.762
1	2 × 10	58	10	56	RG, rlx	3	19	96.4	610.5	0.089	ML	10296.0	4728.4	0.367
1	F20	63	6	63	RG	3	16	65.4	454.1	0.111	ML	212.4	4.7	0.560
1	F45	57	7	57	RG	3	16	56.7	349.3	0.000	ML	281.8	91.8	0.929
1	F12	57	8*	52	RG	3	8	43.3	311.1	0.077	ML	189.9	43.6	0.746
2	51 × 35	11	9	10	RG	1	0	51.7	8.4	0.000	ML	70.5	2.7	0.917
2	2 × 10	4	6	4	RG, rlx	1	0	42.4	0.2	0.250	ML	52.0	1.1	1.000
2	F20	8	4	8	RG	3	2	28.7	50.1	0.375	ML	133.6	70.7	0.803
2	F45	11	3	11	RG	1	0	41.0	381.6	0.455	ML	5103.9	4951.7	0.033
2	F12	10	4	9	RG, rlx	3	1	37.1	312.8	0.889	ML	10033.2	9689.3	0.010
3	51 × 35	48	8	48	RGFO	3	1	51.6	21.6	0.875	MLFO	77.4	19.1	0.981
3	2 × 10	49	10	49	RGFO	3	28	100.0	351.4	0.469	MLFO	210.1	48.4	0.965
3	F20	49	4	49	RG	3	3	45.4	356.6	0.449	ML	5086.8	22.1	0.928
3	F45	50	7	50	RG	3	5	48.8	159.2	0.560	ML	10098.9	9689.3	0.741
3	F12	69	5	68	RG	3	22	53.1	621.0	0.235	ML	5425.1	4808.3	0.605
4	51 × 35	40	10	40	RGFO	3	1	60.8	21.6	0.500	ML	99.9	0.8	0.994
4	2 × 10	31	10	31	RG	3	2	71.8	166.9	0.645	ML	269.2	106.0	0.336
4	F20	43	3	43	RGFO, rlx	3	14	94.7	68.7	0.465	ML	238.2	282.3	0.936
4	F45	55	4	55	RG	3	9	73.4	429.1	0.236	ML	274.8	223.9	0.549
4	F12	40	6	37	RG	3	2	57.0	18.7	0.162	ML	107.8	26.4	0.937
5	51 × 35	26	9	25	RGFO	1	0	61.9	7.1	0.000	ML	94.0	25.0	0.984
5	2 × 10	31	5	29	RG	3	2	53.1	311.0	0.103	ML	228.1	61.6	0.095
5	F20	36	5	36	RG	3	6	55.4	93.5	0.083	ML	145.9	25.5	0.711
5	F45	28	4	27	RG	3	6	49.2	88.9	0.259	ML	112.9	21.8	0.612
5	F12	32	4	32	RG	3	1	49.3	63.5	0.031	ML	158.4	62.1	0.939
6	51 × 35	48	8	47	RGFO	3	22	58.0	7.2	0.468	ML	74.6	0.8	0.995
6	2 × 10	60	10	60	RG	3	24	65.4	621.0	0.850	ML	309.4	22.7	0.650
6	F20	55	6	55	RG	3	18	42.8	210.8	0.073	ML	100.3	12.4	0.823
6	F45	61	5	61	RG	3	14	68.4	78.5	0.410	ML	230.8	63.6	0.683
6	F12	64	6	63	RG	3	15	47.0	310.9	0.286	ML	155.7	245.5	0.077
7	51 × 35	53	7	53	RGFO	3	8	67.0	44.3	0.943	MLFO	104.5	29.8	0.947
7	2 × 10	58	9	58	RG, rlx	3	42	94.8	202.7	0.138	ML	5523.7	4923.5	0.547
7	F20	61	4	59	RG	3	9	30.0	378.0	0.407	ML	134.6	27.1	0.099
7	F45	71	4	71	RG	3	20	44.8	374.6	0.239	ML	155.9	39.3	0.661
7	F12	56	8*	54	RG	3	13	54.0	27.3	0.167	ML	75.2	8.8	0.865
8	51 × 35	33	10	33	RGFO	3	2	48.1	38.9	0.545	ML	105.8	5.9	0.915
8	2 × 10	33	10	33	RG	3	12	68.8	473.6	0.212	ML	379.7	103.4	0.686
8	F20	37	4	37	RG	3	7	78.1	293.6	0.324	ML	332.9	74.2	0.367
8	F45	36	7	36	RG	3	2	33.4	619.5	0.278	ML	174.1	230.1	0.081
8	F12	34	6	22	RG	1	0	32.8	11.8	0.000	ML	51.1	28.3	0.901
9	51 × 35	16	8	16	RGFO	3	1	57.8	296.7	0.063	MLFO	298.2	39.5	0.942
9	2 × 10	19	10	19	RG	3	7	35.1	78.9	0.053	ML	142.2	49.2	0.029
9	F20	13	5	13	RG	3	1	42.1	144.9	0.385	ML	88.1	35.1	0.362
9	F45	14	5	14	RG, rlx	3	2	39.0	60.1	0.143	ML	78.7	17.3	0.877
9	F12	13	4	13	RG	1	0	38.8	34.8	0.154	ML	90.3	30.4	0.269
10	51 × 35	55	8	55	RG, rlx	3	7	65.7	51.1	0.945	ML	143.0	48.8	0.947
10	2 × 10	79	10	79	RG	3	26	62.0	621.3	0.190	ML	10500.5	5000.5	0.657
10	F20	72	5	71	RG	3	16	52.2	312.6	0.423	ML	157.6	37.6	0.790
10	F45	59	4	59	RG	3	9	52.9	191.5	0.169	ML	5293.2	4856.2	0.856
10	F12	63	6	63	RG	3	24	50.8	307.6	0.651	ML	239.2	64.4	0.597

LG, linkage groups; LOD, log of the odds; RG, regression; ML, maximum likelihood.

a Grouping LOD is the threshold passed by all markers in at least one test of independence with other markers in the group. For two values marked by an asterisk, the group was formed from smaller groups passing the LOD threshold indicated.

b RG indicates default regression mapping settings in JoinMap; “rlx” indicates relaxed linkage thresholds for regression mapping. RGFO uses a fixed order of anchor markers established by ML mapping and agreement of ML and RG marker orders.

c MLFO uses a fixed order of anchor markers.

The ML method yields better fitting maps, in most but not all cases; the maximum nearest-neighbor fit statistic per linkage group exceeds map length in only three of 50 cases. However, the ML maps are invariably longer than those produced by the regression method (Table 6). Nine of the 50 ML maps have lengths of 5000+ or 10,000+ cM, owing to markers that have recombination frequencies with flanking markers of 0.5 in one parent but not in the other parent (the ML map is an average of the two parental maps). Often, these markers are segregating from only one parent (mating types, *np* × *nn* or *ll* × *lm* in Table 1). Excluding these excessively long maps, the average ML map is still three times longer than the corresponding regression map. The orders of markers produced by these maps are, nevertheless, strongly supported by the simulated annealing algorithm implemented in JoinMap (Jansen *et al.* 2001), except for closely linked markers. Agreement between the order of markers on RG and ML maps is assessed by the proportion of variance explained by a linear fit (*r*^2^) of RG marker rank order on ML marker rank order. These *r*^2^ values range from 0.01 to 1.0, with 24 of 50 exceeding 0.75 but 8 falling below 0.1 (Table 6)

Distortion of Mendelian segregation ratios is widespread over these maps, as observed in previous studies (Launey and Hedgecock 2001; Li and Guo 2004; Sauvage *et al.* 2010; Plough and Hedgecock 2011; Plough 2012; Guo *et al.* 2012). The extent of this distortion is represented in Table 6 and Table 7 by the proportion of markers on each linkage group with a χ^2^ goodness-of-fit test probability less than or equal to 0.01. In the vast majority of cases, markers with distorted segregation ratios are clustered on linkage maps, again, as observed in previous studies (*op. cit*.). The mean proportion of distorted markers on a linkage group, 0.31, does not vary among families but does vary among linkage groups (*F*_9,36_ = 2.404, *P* = 0.03), ranging from a low of 0.034 on LG 1 to highs of 0.56 on LG 10 and 0.61 on LG 3. There is no significant relationship between proportions of markers distorted and either map lengths or maximum nearest-neighbor fit statistics across linkage groups; but certain distorted markers clearly do contribute to poor fit and inflated map lengths (examples given below).

**Table 7:** Summary statistics for regression and maximum likelihood linkage maps made with bi-parentally inherited, framework markers, in five families of Pacific oyster

LG	Family	No. Markers	No. Markers Used	RG Type*^a^*	Map Round	No. Markers Forced	RG Length, cM	RG Max. NNFit	Prop. Markers distorted	ML Type*^b^*	ML Length, cM	ML Max. NNFit	RG *v*. ML *r^2^*
1	51 × 35	34	32	RG	1	0	58.7	2.7	0.000	ML	62.8	0.8	0.996
1	2 × 10	30	22	RG	2	0	57.5	1.9	0.000	ML	86.2	23.1	0.993
1	F20	19	16	RG	2	0	59.8	22.5	0.000	ML	68.1	2.9	0.940
1	F45	21	16	RG	1	0	39.3	3.3	0.000	ML	34.9	1.1	0.996
1	F12	15	14	RG	1	0	73.4	21.4	0.000	ML	119.0	3.5	0.993
2	51 × 35	5	5	RG, rlx	1	0	59.3	2.8	0.000	ML	74.5	5.5	1.000
2	2 × 10	4	4	RG, rlx	1	0	42.4	0.2	0.250	ML	52.0	1.1	1.000
2	F20	3	3	RG	3	1	65.3	10.5	0.667	ML	85.7	0.5	1.000
2	F45	7	3	RG	1	0	22.7	0.6	0.000	ML	28.1	2.7	1.000
2	F12	3	3	RG	1	0	20.9	0.1	0.667	ML	24.4	1.2	1.000
3	51 × 35	38	38	RGFO	1	0	50.5	11.5	1.000	ML	80.1	2.0	0.978
3	2 × 10	31	31	RGFO	3	4	57.8	161.2	0.613	MLFO	160.8	6.4	0.706
3	F20	18	15	RG	1	0	61.0	11.9	0.133	ML	86.8	5.8	0.993
3	F45	21	21	RG	1	0	50.0	21.0	0.762	ML	96.6	2.0	0.915
3	F12	28	22	RG	3	1	75.9	10.9	0.136	ML	184.3	95.9	0.979
4	51 × 35	40	40	RGFO	3	1	60.8	21.6	0.500	MLFO	108.7	5.5	0.997
4	2 × 10	23	23	RG	1	0	56.3	2.1	0.522	ML	60.2	0.5	0.995
4	F20	13	12	RG	1	0	50.6	91.7	0.750	ML	90.1	3.3	0.843
4	F45	18	17	RG	3	1	68.5	33.3	0.176	ML	111.0	5.7	0.947
4	F12	11	10	RG	1	0	46.6	5.9	0.200	ML	64.2	5.6	0.988
5	51 × 35	18	18	RG	1	0	58.9	5.3	0.000	ML	61.5	1.9	0.996
5	2 × 10	14	14	RG	2	0	46.2	6.8	0.071	ML	67.7	8.0	0.862
5	F20	10	10	RG	1	0	78.6	5.8	0.000	ML	112.3	9.5	0.994
5	F45	9	8	RG	1	0	50.5	14.3	0.500	ML	73.7	1.2	0.953
5	F12	12	11	RG	1	0	49.0	4.4	0.000	ML	53.8	4.9	0.986
6	51 × 35	46	45	RG	1	0	58.8	6.3	0.467	ML	74.9	1.1	0.981
6	2 × 10	30	28	RG	3	1	61.7	41.5	1.000	ML	139.6	5.1	0.849
6	F20	20	19	RG	3	7	96.8	76.5	0.105	ML	151.6	25.5	0.622
6	F45	13	10	RG	1	0	45.2	92.8	0.300	ML	80.6	4.1	0.360
6	F12	27	24	RG	1	0	60.7	5.0	0.125	ML	79.6	21.6	0.880
7	51 × 35	49	49	RG	2	0	59.1	59.4	1.000	ML	107.7	2.2	0.944
7	2 × 10	25	16	RG	1	0	50.1	2.2	0.000	ML	56.8	0.8	0.999
7	F20	21	18	RG, rlx	3	9	129.7	58.0	0.500	ML	174.9	8.2	0.309
7	F45	25	16	RG	2	0	77.9	10.0	0.000	ML	117.4	24.9	0.962
7	F12	17	17	RG	1	0	50.6	1.7	0.294	ML	60.4	6.2	0.990
8	51 × 35	26	23	RG	1	0	51.2	3.0	0.652	ML	58.7	1.2	0.994
8	2 × 10	27	22	RG	3	2	56.4	2.4	0.000	ML	70.2	1.7	0.976
8	F20	13	13	RG	1	0	94.1	22.8	0.308	ML	167.3	5.3	0.714
8	F45	13	13	RG	1	0	35.8	6.4	0.077	ML	111.4	50.1	0.956
8	F12	13	13	RG	1	0	69.0	22.9	0.077	ML	120.6	24.0	0.957
9	51 × 35	6	6	RGFO	1	0	52.7	0.8	0.000	ML	70.0	6.4	1.000
9	2 × 10	10	10	RG	1	0	44.2	1.9	0.000	ML	49.1	1.0	1.000
10	51 × 35	44	42	RGSO	3	6	50.2	55.5	1.000	ML	90.5	0.7	0.932
10	2 × 10	36	24	RG	1	0	55.1	2.0	0.000	ML	134.4	63.2	0.952
10	F20	24	22	RG	2	0	58.3	376.2	0.364	ML	5171.7	4888.3	0.092
10	F45	25	21	RG	1	0	40.6	3.4	0.000	ML	52.1	2.1	0.994
10	F12	19	17	RG	3	1	43.6	46.7	0.588	ML	84.9	2.8	0.174

a RG indicates default regression mapping settings in JoinMap; “rlx” indicates relaxed linkage thresholds for regression mapping. RGFO uses a fixed order of anchor markers established by ML mapping and agreement of ML and RG marker orders; RGSO uses the order of anchor markers as a starting order in the regression mapping analysis.

b MLFO uses a fixed order of anchor markers.

Having observed effects of markers segregating from only one parent on map orders and lengths, we proceed with linkage maps of framework markers segregating from both parents (mating types *hk × hk*, *ef × eg*, and *ab × cd* in Table 1). For five framework maps, we exclude obviously poorly fitting markers, which are microsatellite DNA markers in seven of 17 cases. Thresholds for regression mapping are relaxed in only three of 47 cases (Table 7; the total number of maps is reduced from 50 because families F12, F20, and F45 had too few two biparentally segregating markers on LG 9 for map construction). Only 11 of 47 regression-method maps are third-round maps, which force an average of 3.2 markers onto those maps. Maximum nearest-neighbor fit still ranges widely, from 0.1 cM to 615.5 cM, but only 10 of 47 fit statistics exceed the estimated lengths of their LGs. As in the analyses of all markers, ML maps generally fit the data on biparentally segregating markers better than do RG maps, although the ML maps are still longer in each case. Excluding two maps that exceed 5000 cM, the average ML map is 1.6 times as long as its regression-method counterpart. The *r*^2^ for the linear fit of RG marker order to ML marker order ranges from 0.09 to 1.0, with 29 of 47 being 1.0 and 36 exceeding 0.75. These framework maps were used to make consensus maps (see *Consensus linkage maps*).

**Figure 2:**
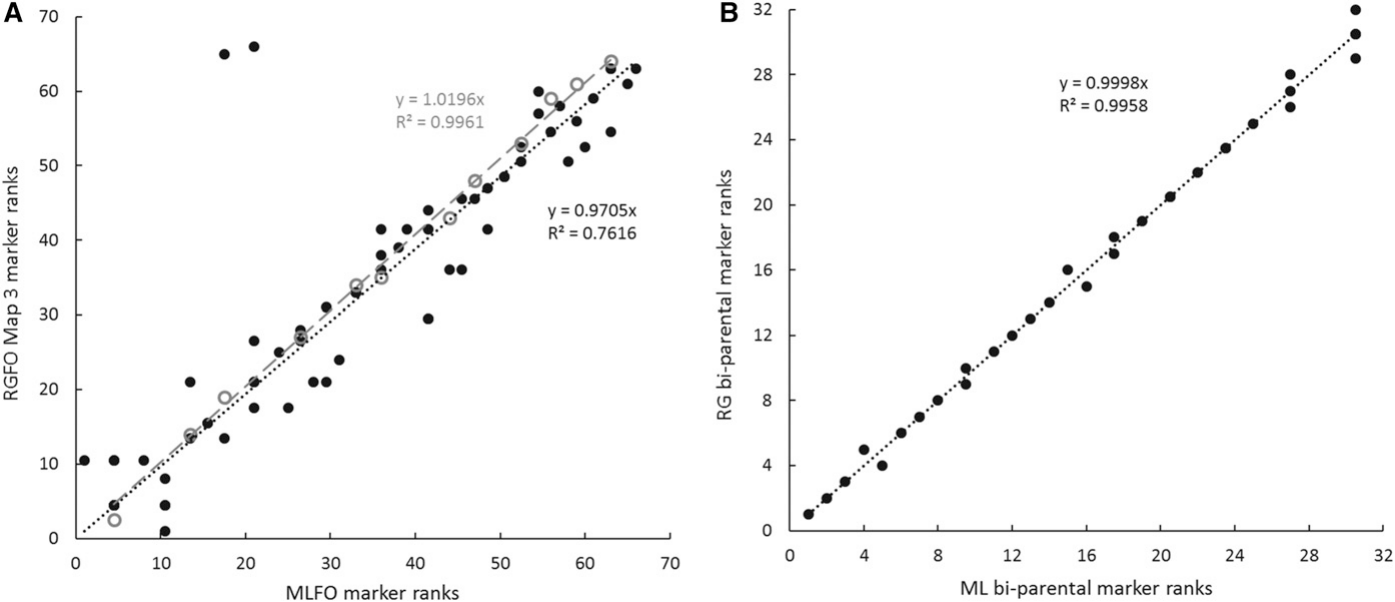
Regression of regression (RG) marker order on maximum likelihood (ML) marker order for linkage group 1 of family 51 × 35, from (A) fixed-order maps (RGFO, MLFO; black, filled circles and black, dotted trend line [*y* = 0.9705*x*], for all 66 markers; gray, open circles and gray, dashed trend line [*y* = 1.0196*x*], for 12 anchor loci used to fix order) and (B) maps of 32 framework markers with no fixed-order.

**Figure 3:**
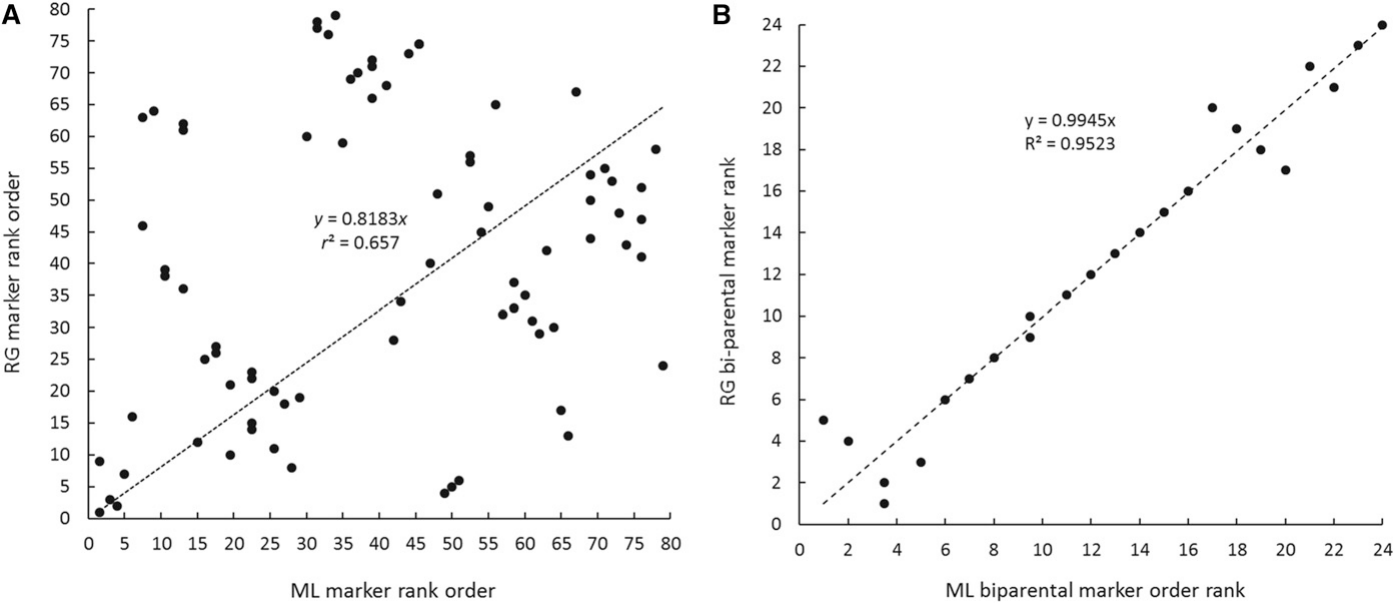
Regression of regression (RG) marker order on maximum likelihood (ML) marker order for linkage group 10 of family 2 × 10, from (A) maps with 79 markers and (B) maps with 24 framework markers.

The plausibility of marker positions for two ML maps, for all markers and for biparentally segregating framework markers, are presented in supplementary information (Figure S1 and Figure S2), to illustrate factors influencing map lengths and marker orders. In the first example, LG 1 for family 51 × 35, segregation ratios conform to Mendelian expectations, so selection is not a factor. However, markers segregating from only one parent cause moderately large nearest-neighbor fits (*cf*. Figure S1A and Figure S1B with Figure S1C) and, at the termini of both parental maps, expansions of map lengths from about 70 cM to more than 200 cM. Still, agreement in the rank order of 66 markers between the RG and ML maps is fairly good, with only a few outliers (Figure 2A). Regression of RG on ML marker order is greatly improved for 32 framework markers (Figure 2B). In the second example (79 markers grouped for LG 10 of family 2 × 10), severely distorted Mendelian ratios are associated with large jumps in length and regions of very poor fit on ML parental maps (Figure S2A and Figure S2B). Single-parent segregations also contribute to regions of poor fit and length expansion. Regression of RG on ML marker orders is quite poor for all 79 markers (Figure 3A). In attempting to construct the framework marker map, 12 markers with highly distorted segregation ratios had to be removed to achieve reasonable congruence of the remaining 24 marker orders on the RG and ML framework maps (Figure 3B). Still, this reduced ML framework map, though unaffected by selection, has one region of poor fit (Figure S2C) and is 2.4× as long as the comparable RG map (Table 7).

### Differences in recombination between sexes

**Figure 4:**
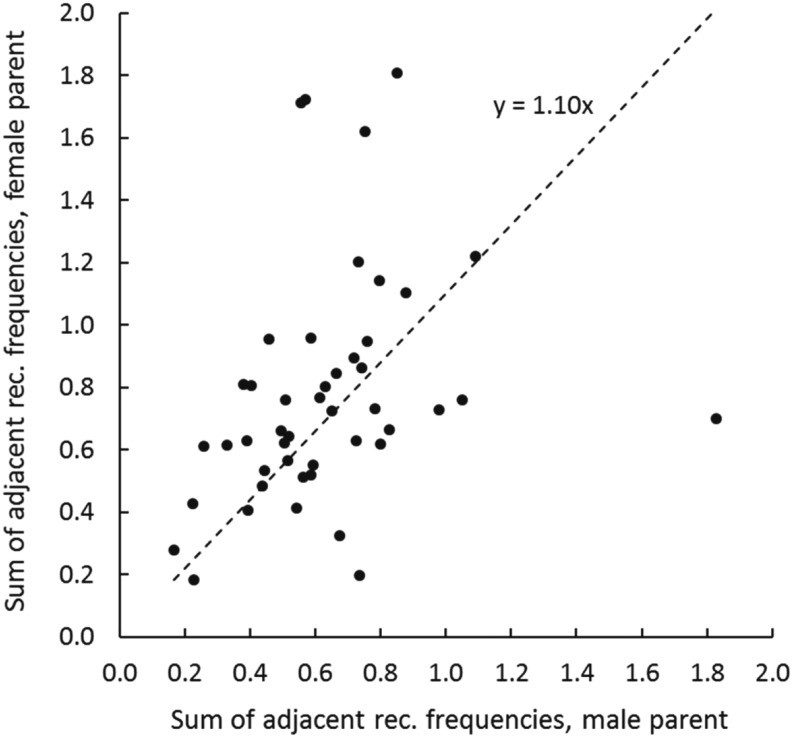
Comparison of the sums of adjacent recombination frequencies from 46 framework maximum likelihood linkage maps or male and female parents of five Pacific oyster mapping families.

To assess differences in recombination between males and females, we plot sums of adjacent recombination frequencies—the parameter optimized in ML mapping—for maternal against paternal ML framework linkage maps (Figure 4). For 32 maps, the sum of adjacent recombination frequencies for the female map exceeds that for the male map, while for 14 maps, the opposite is true; however, assuming an intercept of zero, the slope of the regression, is not significantly different than 1.0.

### Consensus linkage maps

To build a consensus linkage map for the Pacific oyster, we used individual family ML framework maps, with the exceptions described in *Materials and Methods*. The final consensus linkage map has 656 markers, including 49 previously mapped microsatellite DNA markers, and spans, after adjustment, 890 cM (Table S7).

## Discussion

More than 1100, coding SNPs are placed on a second-generation genetic linkage map for the Pacific oyster *Crassostrea gigas*, along with 66 microsatellite DNA markers to establish coherence with first-generation maps. On average, the map has a spacing of 1 cM and covers 86% of a genome that is estimated, here, to be ∼616 cM in total length. This observed map length corresponds well with both a cytological estimate of map length in the eastern oyster *Crassostrea virginica* (1.2 crossovers per bivalent × 50 cM × 10 chromosomes = 600 cM; Longwell *et al.* 1967) and with genome-size estimates for the Pacific oyster, from either flow cytometry or assembly of DNA sequences (637 Mb and 559 Mb, respectively; Zhang *et al.* 2012), if the 1 cM ≈ 1 Mb rule of thumb from human genetics (http://ghr.nlm.nih.gov/glossary=centimorgan) applies to the oyster.

In contrast to what Hubert and Hedgecock (2004) reported, no significant differences in map distances are detected across 46 male- and female-parent maps, suggesting no marked difference in underlying male and female recombination rates in this much larger data set. On the other hand, the distribution of SNPs among parents appears to reflect both the breeding history of the mapping families used (F_2_ *vs.* wild-caught) and an ascertainment bias stemming from the initial identification of SNPs fixed for different alleles in the inbred parent lines of the 51 × 35 F_2_ family.

This second-generation map is a substantial improvement over previously published, first-generation maps for this species, which were either low-density maps based on microsatellite DNA markers or on small numbers of SNPs or higher-density maps based on dominant, largely nontransferable AFLP markers. A consensus map based on three families and 100 microsatellite DNA markers (Hubert and Hedgecock 2004; Hubert *et al.* 2009) had average lengths of 616 cM and 770 cM for male- and female-based maps, respectively, average spacing of 8−10 cM and genome coverage of 70–80%. Li and Guo (2004) constructed male and female maps with dominant AFLP markers; the male map had 96 markers in 10 linkage groups, covering 758 cM with an average spacing of 8.8 cM, whereas the female map had 119 markers in 11 linkage groups, covering 1031 cM with an average spacing of 9.5 cM. Later, Guo *et al.* (2012; see also Zhong *et al.* 2014) published another map based on a single, full-sib family typed for 64 genomic microsatellite DNA markers, 42 EST-associated microsatellite DNA markers, and 320 dominant AFLP markers; this map spanned 558 cM, had an average spacing of 1.3 cM, and covered 95% of the genome. Sauvage *et al.* (2010) built a consensus map of 51 microsatellite markers and 29 EST-derived SNPs typed in three F_2_ families, which spanned 1016 cM, had an average spacing of 12.7 cM, and covered 73.6% of the genome. Most first-generation maps are much longer than the second-generation map reported here and, of course, have much lower densities of markers. All of these previous reports note significant distortion of segregation ratios for a substantial proportion of markers, rendering mapping difficult in some cases (Sauvage *et al.* 2010).

### Implications for genome scaffold assembly

Perhaps, the most surprising finding in this study was the frequency with which markers on the same genome scaffold map to different LGs. Nearly 40% of genome scaffolds with two or more SNPs map to different linkage groups, which strongly suggests that a substantial fraction of the genome scaffolds reported by Zhang *et al.* (2012) are incorrectly assembled. The likelihood of misassembly appears to rise with scaffold length, and additional statistical comparisons of inter-marker distances and numbers of ambiguous bases between adjacent SNPs support this inference. Adjacent SNPs mapping to different LGs are 4.7 times farther apart than adjacent SNPs mapping to the same LG, whereas sequences between adjacent SNPs that do or do not map to the same LG have median numbers of 11 and 25,161 ambiguous bases, respectively. Because the minimum number of breakpoints required to explain the order of mapped SNPs is found in 58 of 60 informative cases, our data suggest that misassembled scaffolds comprise large blocks (contigs or super-contigs, see Figure 1 in Zhang *et al.* 2012) that map to different linkage groups and are separated by long stretches of ambiguous bases. These large scaffolds should be shattered at potential breakpoints and reassembled using linkage information or long-range DNA sequences. That 50% of the genome assembly is found in the largest 401 scaffolds (N50 size of 401.3 kb; Zhang *et al.* 2012) will likely prove to be an overestimate.

This conclusion about scaffold assemblies contrasts sharply with the fact that 95% of ESTs were successfully mapped to the draft genome assembly (see Table S6 in Zhang *et al.* 2012). Evidently, at the level of contigs and genes, the assembly of the Pacific oyster genome is quite complete and useful, but at the level of scaffolds, the assembly contains many errors. Because the ordering of contigs and genes within scaffolds may not always be accurate, inferences about the clustering of gene families (*e.g.*, Paps *et al.* 2015) should be regarded as provisional until the assembly of genome scaffolds is either confirmed or improved. High density linkage maps are presently being constructed, using genotyping-by-sequencing methods (Elshire *et al.* 2011); these maps should provide critical information for improving future assemblies of the Pacific oyster genome, as they have in other species (*e.g.*, Dalloul *et al.* 2010; Dodgson *et al.* 2010).

### Uncertainties in linkage maps

In the course of this study, three factors emerged as evident causes of discrepancies in map orders and lengths between RG and ML methods and among families. The first, mentioned above and illustrated by two examples (Figure S1, Figure S2, Figure 2, and Figure 3), is the effect of markers segregating from only one parent, which consistently contribute to inflated ML map lengths and poor agreement between RG and ML maps. Across all 47 linkage groups, the general effect of single-parent markers is reflected in the average improvement in agreement of RG and ML marker orders between maps made with all markers and maps made only from bi-parentally segregating framework markers (mean *r*^2^ for all-marker maps, 0.67; mean *r*^2^ for framework maps, 0.85; paired sample *t* = 3.17, *P* = 0.0014). For this reason, only framework maps are merged into consensus linkage maps.

The second factor causing uncertainty in map construction is distortion of Mendelian inheritance ratios, owing to strong viability selection, as documented in several previous studies of oysters (Bierne *et al.* 1998; Launey and Hedgecock 2001; Plough and Hedgecock 2011; Plough 2012). In this study, 45 of 50 linkage groups show evidence of viability selection (Table 2), yet the effect of viability selection on map construction is not a general one. Across all linkage groups, we could find no significant relationship between the proportions of markers distorted and the lengths of maps, the fit of maps to data, or the agreement of RL and ML marker orders. Furthermore, counterexamples to a general effect of viability selection on linkage mapping are evident—linkage groups with no evidence of selection but with uncertainties in map orders and lengths (*e.g.*, Figure S1 and Figure 2) or linkage groups with most or even all markers showing distorted segregation ratios but having well-behaved maps (LG 3, LG 7, and LG 10 for family 51 × 35, Table 6 and Table 7). On the other hand, distorted markers do often contribute to poor fit and extended map lengths (Figure S2 and Figure 3). This is especially the case, if markers segregating from one or the other parent fall into regions of segregation distortion.

Mode of selection appears to make a difference, because massively distorted but well-behaved LGs occur only in the F_2_ families, in which associative overdominance—the apparent advantage of heterozygotes, owing to selection against recessive deleterious mutations linked to alternative homozygotes—is a consequence of their breeding history, as is the excess of *hk*×*hk* mating types in these families (Table 1; Plough and Hedgecock 2011). For example, on the ML framework map for LG 10 in family 51 × 35, all 42 markers are highly significantly distorted, but nearest-neighbor fit averages only 0.085 cM and the RG and ML maps are highly correlated (*r*^2^ = 0.93; Table 7). The relative fitness of heterozygotes compared to the most abundant homozygotes across the ML map for this linkage group averages 2.03, reaching a peak at 10.3, where genotypic proportions for SNP 1385-A193329G are zero *hh*, 103 *hk*, and 5 *kk*. In this case, the symmetry of the selection on parental types appears not to affect the fit of observed and calculated recombination frequencies and marker orders. In contrast, a framework ML map of 36 markers for the same linkage group, LG 10, in family 2 × 10, has two blocks of distorted markers (12 markers altogether, which had to be removed to produce the framework map recorded in Table 7 and Figure 3), but nearest-neighbor fit averages 281 cM and orders of markers on the RG and ML maps are poorly correlated (*r*^2^ = 0.61). The average relative fitness of heterozygotes compared to the most abundant homozygotes at each marker across this ML map is 0.75, reaching a nadir of ∼0.3, where one homozygote disappears entirely. Here, the gross asymmetry and partial dominance of viability selection is clearly associated with inflation of ML map distances and incongruence of RG and ML marker orders. Thus, the number, location, and phase of loci under viability selection produce idiosyncratic effects on map lengths and marker orders. Detailed analyses of the location and effects of viability mutations in the G_0_ families are reported elsewhere (L. V. Plough, G. Shin, and D. Hedgecock, unpublished data).

Distortion of Mendelian segregation ratios is commonly observed in mapping studies, especially in plants and marine molluscs, although the phenomenon may generally be underreported, because distorted markers often are discarded from data sets before linkage analysis. Distortion of segregation ratios can arise in interspecific or intersubspecific crosses, because of Dobzhansky-Muller incompatibilities (*e.g.*, Fishman *et al.* 2001; Wang *et al.* 2005; Foley *et al.* 2013), but our focus, here, is on distortions of segregation ratios arising from genotype-dependent mortality of progeny from intraspecific crosses of Pacific oysters (Launey and Hedgecock 2001; Plough and Hedgecock 2011). Viability loci causing early mortality in oysters appear to act independently of one another, as no statistically significant epistatic interactions are detected (Plough and Hedgecock 2011; Plough 2012; L.V. Plough, G. Shin, and D. Hedgecock, unpublished data).

Advice on what to do about segregation distortion in linkage mapping studies ranges from discard distorted markers, to exercise great caution in interpreting results from commonly used computer packages (Liu 1998), to include such markers, so as to retain genetic and potentially biologically important information (Shah *et al.* 2014; Truong *et al.* 2014). Theoretical studies on how segregation distortion can bias estimates of recombination date back to Bailey (1949) and Allard and Alder (1960) but yield few generalizations regarding the practice of linkage mapping, particularly now, when dense linkage maps are enabled by high-throughput DNA sequencing. Estimates of recombination are affected by the form of selection (allelic *vs.* zygotic), by the dominance of viability genes, and by the dominance of markers (Lorieux *et al.* 1995; Liu 1998), which suggests that impacts of segregation distortion are likely to vary within and among linkage maps, owing to variation in the underlying modes of selection. Hackett and Broadfoot (2003) concluded that segregation distortion does not substantially impact map length or marker order, but they simulated only one distorted marker per linkage group, in a doubled-haploid mapping population, which may underestimate the impact of segregation distortion in other mapping populations or species with large numbers of viability loci, such as the oyster. Marker density also appears to be a factor, for example, marker orders in low-density, first-generation linkage maps for the Pacific oyster were reasonably consistent across families and studies (Plough and Hedgecock 2011; Plough 2012; Guo *et al.* 2012), despite widespread distortion of segregation ratios.

Efficient estimators of maps that take into account distortion or even simultaneously estimate fitness parameters are either computationally burdensome for dense maps or, when made computationally efficient, have so far been developed only for mapping populations common in plant genetics, *i.e.*, F_2_, doubled-haploid, BC, RIL, and multiparent advanced generation intercross (*i.e.*, MAGIC) populations (Wu *et al.* 2008; Zhu *et al.* 2007; Zhu and Zhang 2007; Lorieux 2012; Shah *et al.* 2014). In this study, we have left distorted markers in all analyses, discarding them only from the minority of framework maps in which they prevent a reasonable fit between observed and expected recombination rates or good agreement of marker orders from the regression and maximum likelihood mapping methods. For future linkage or QTL mapping studies with oysters, doubts about marker orders and distances, owing to selection and segregation distortion, can largely be eliminated by taking samples for genotyping in the mid to late larval stages (in addition to the life stage or time-point of interest), since much of the selective mortality occurs later, during metamorphosis (Plough and Hedgecock 2011).

A third factor likely contributing to discrepancies in map order and lengths is genotyping error (Hackett and Broadfoot 2003). Microsatellite genotypes for families F12, F20, and F45 were scored in duplicate reactions for at least 5% of individuals and an average of 17 markers, in each family (L. V. Plough, G. Shin, and D. Hedgecock, unpublished data), revealing an average error rate of 2.5%. Such an error rate has a small effect on map accuracy, when marker densities are low (∼10 cM), as in previous linkage maps for the Pacific oyster based on microsatellite DNA markers (Plough and Hedgecock 2011; Plough 2012; L.V. Plough, G. Shin, and D. Hedgecock, unpublished data), but can impair the ordering of markers, when the density of markers is 1-2 per cM, as in this study (Hackett and Broadfoot 2003; Ball *et al.* 2010). That microsatellite markers frequently have poor nearest-neighbor fits is most likely explained by their relatively large genotyping error in the context of dense, second-generation SNP maps. The genotyping error of SNP markers, on the other hand, was extremely low (zero observed in 12,937 trials). Still, an observed frequency of zero is compatible with an error rate as high as 3 in 12,937, which yields a 5% chance of observing no errors in 12,937 trials of a binomial distribution. Given this potential error rate and the large number of genotypes determined in this study, nearly 172 thousand, the total number of genotyping errors could have ranged from 19 to 61 (99.9% confidence limits), with an average of 40 errors, and therefore could have contributed to otherwise unexplained problems in mapping.

### A second-generation consensus linkage map for the Pacific oyster

The consensus linkage map made with MergeMap (Wu *et al.* 2011) appears too long (890 cM), compared to the estimated length of the genetic map (616 cM) and the size of the genome (637 Mb) estimated by flow cytometry (Zhang *et al.* 2012). Still, this map likely represent the best consensus ordering of markers and is presented here for future reference (Table S7).

## 

## Supplementary Material

Supporting Information

## References

[bib1] Allard A. W. Alder H. L., 1960 The effect of incomplete penetrance on the estimation of recombination values. Heredity 15: 263–282.

[bib2] Applebaum S. L. Pan T.-C. F. Hedgecock D. Manahan D. T., 2014 Separating the nature and nurture of energy allocation in response to global change. Integr. Comp. Biol. 54: 284–295. 24907199 10.1093/icb/icu062

[bib3] Bailey N. T. J., 1949 The estimation of linkage with differential viability, II and III. Heredity 3: 220–228. 10.1038/hdy.1949.13 18143388

[bib4] Ball A. D. Stapley J. Dawson D. A. Birkhead T. R. Burke T., 2010 A comparison of SNPs and microsatellites as linkage mapping markers: lessons from the zebra finch (*Taeniopygia guttata*). BMC Genomics 11: 218. 20359323 10.1186/1471-2164-11-218 PMC2864244

[bib5] Bierne N. Launey S. Naciri-Graven Y. Bonhomme F., 1998 Early effect of inbreeding as revealed by microsatellite analyses on *Ostrea edulis* larvae. Genetics 148: 1893–1906. 9560403 10.1093/genetics/148.4.1893 PMC1460075

[bib6] Bishop D. T. Cannings C. Skolnick M. Williamson J. A., 1983 The number of polymorphic DNA clones required to map the human genome in Statistical Analysis of DNA Sequence Data, edited by Weir B. S. Marcel Dekker, New York.

[bib7] Chakravarti A. Lasher L. K. Reefer J. E., 1991 A maximum likelihood for estimating genome length using genetic linkage data. Genetics 128: 175–182. 2060775 10.1093/genetics/128.1.175 PMC1204446

[bib8] Chevreux B. Pfisterer T. Drescher B. Driesel A. J. Muller W. E. G., 2004 Using the miraEST assembler for reliable and automated mRNA transcript assembly and SNP detection in sequenced ESTs. Genome Res. 14: 1147–1159. 15140833 10.1101/gr.1917404 PMC419793

[bib9] Curole J. P. Hedgecock D., 2007 Bivalve genomics: complications, challenges, and future perspectives, pp. 525–543 in Aquaculture Genome Technologies, edited by Liu Z. J. Blackwell Publishing, Ames, Iowa.

[bib10] Dalloul R. A. Long J. A. Zimin A. V. Aslam L. Beal K., 2010 Multi-platform next-generation sequencing of the domestic turkey (*Meleagris gallopavo*): Genome assembly and analysis. PLoS Biol. 8: e1000475. 20838655 10.1371/journal.pbio.1000475 PMC2935454

[bib11] Dégremont L. Bédier E. Boudry P., 2010 mortality of hatchery-produced Pacific oyster spat (*Crassostrea gigas*). II. Response to selection for survival and its influence on growth and yield. Aquaculture 299: 21–29.

[bib12] Dodgson J. B. Delany M. E. Cheng H. H., 2010 Poultry genome sequences: Progress and outstanding challenges. Cytogenet. Genome Res. 134: 19–26. 21335957 10.1159/000324413

[bib13] Doerge R. W., 2002 Mapping and analysis of quantitative trait loci in experimental populations. Nat. Rev. Genet. 3: 43–52. 11823790 10.1038/nrg703

[bib14] Elshire R. J. Glaubitz J. C. Sun Q. Poland J. A. Kawamoto K., 2011 A robust, simple genotyping-by-sequencing (GBS) approach for high diversity species. PLoS One 6: e19379. 21573248 10.1371/journal.pone.0019379 PMC3087801

[bib15] Food and Agriculture Organization (FAO), 2015 World aquaculture production of fish, crustaceans, molluscs, etc., by principal species in 2013. Available at: ftp://ftp.fao.org/fi/stat/summary/a-6.pdf. Accessed August 10, 2015.

[bib16] Fishman L. Kelly A. J. Morgan E. Willis J. H., 2001 A genetic map in the *Mimulus guttatus* species complex reveals transmission ratio distortion due to heterospecific interactions. Genetics 159: 1701–1716. 11779808 10.1093/genetics/159.4.1701 PMC1461909

[bib17] Foley B. R. Rose C. G. Rundle D. E. Leong W. Edmands S., 2013 Postzygotic isolation involves strong mitochondrial and sex-specific effects in *Tigriopus californicus*, a species lacking heteromorphic sex chromosomes. Heredity 111: 391–401. 23860232 10.1038/hdy.2013.61 PMC3806025

[bib18] Guo X. Li Q. Wang Q. Z. Kong L. F., 2012 Genetic mapping and QTL analysis of growth-related traits in the Pacific oyster. Mar. Biotechnol. (NY) 14: 218–226. 21932055 10.1007/s10126-011-9405-4

[bib19] Hackett C. A. Broadfoot L. B., 2003 Effects of genotyping errors, missing values and segregation distortion in molecular marker data on the construction of linkage maps. Heredity 90: 33–38. 12522423 10.1038/sj.hdy.6800173

[bib20] Hedgecock D. Davis J. P., 2007 Heterosis for yield and crossbreeding of the Pacific oyster *Crassostrea gigas*. Aquaculture 272(Suppl. 1)**:** S17–S29.

[bib21] Hedgecock D. Gaffney P. M. Goulletquer P. Guo X. Reece K., 2005 The case for sequencing the Pacific oyster genome. J. Shellfish Res. 24: 429–441.

[bib22] Hershberger W. K. Perdue J. A. Beattie J. H., 1984 Genetic selection and systematic breeding in Pacific oyster culture. Aquaculture 39: 237–245.

[bib23] Hubert S. Hedgecock D., 2004 Linkage maps of microsatellite DNA markers for the Pacific oyster *Crassostrea gigas*. Genetics 168: 351–362. 15454548 10.1534/genetics.104.027342 PMC1448102

[bib24] Hubert S. Cognard E. Hedgecock D., 2009 Centromere-mapping in triploid families of the Pacific oyster *Crassostrea gigas* (Thunberg). Aquaculture 288: 172–183.

[bib25] Jansen J. De Jong A. G. Van Ooijen J. W., 2001 Constructing dense genetic linkage maps. Theor. Appl. Genet. 102: 1113–1122.

[bib26] Lander E. S. Botstein D., 1989 Mapping Mendelian factors underlying quantitative traits using RFLP linkage maps. Genetics 121: 185–199. 2563713 10.1093/genetics/121.1.185 PMC1203601

[bib27] Langdon C. Evans F. Jacobson D. Blouin M., 2003 Yields of cultured Pacific oysters *Crassostrea gigas* Thunberg improved after one generation of selection. Aquaculture 220: 227–244.

[bib28] Launey S. Hedgecock D., 2001 High genetic load in the Pacific oyster *Crassostrea gigas*. Genetics 159: 155–165. 10.1093/genetics/159.1.255 PMC1461778 11560902

[bib29] Li H. Durbin R., 2009 Fast and accurate short read alignment with Burrows-Wheeler Transform. Bioinformatics 25: 1754–1760. 19451168 10.1093/bioinformatics/btp324 PMC2705234

[bib30] Li H. Handsaker B. Wysoker A. Fennell T. Ruan J. 1000 Genome Project Data Processing Subgroup, 2009 The Sequence alignment/map (SAM) format and SAMtools. Bioinformatics 25: 2078–2079. 19505943 10.1093/bioinformatics/btp352 PMC2723002

[bib31] Li L. Guo X. M., 2004 AFLP-based genetic linkage maps of the Pacific oyster *Crassostrea gigas* Thunberg. Mar. Biotechnol. (NY) 6: 26–36. 14564534 10.1007/s10126-003-0001-0

[bib32] Liu B. H., 1998 *Statistical Genomics: Linkage*, *Mapping*, *and QTL Analysis*. CRC Press, Florida.

[bib33] Longwell A. C. Stiles S. S. Smith D. G., 1967 Chromosome complement of the American oyster *Crassostrea virginica* as seen in meiotic and cleaving eggs. Can. J. Genet. Cytol. 9: 845–856. 5588794 10.1139/g67-090

[bib34] Lorieux M., 2012 MapDisto: fast and efficient computation of genetic linkage maps. Mol. Breed. 30: 1231–1235.

[bib35] Lorieux M. Perrier X. Goffinet B. Lanaud C. de León D. G., 1995 Maximum-likelihood models for mapping genetic markers showing segregation distortion. 2. F2 populations. Theor. Appl. Genet. 90: 81–89. 24173787 10.1007/BF00220999

[bib36] Mann R., 1979 Exotic Species in Mariculture. MIT Press, Massachusetts.

[bib37] Paps J. Xu F. Zhang G. Holland P. W. H., 2015 Reinforcing the egg-timer: recruitment of novel Lophotrochozoa homeobox genes to early and late development in the Pacific oyster. Genome Biol. Evol. 7: 677–688. 25631164 10.1093/gbe/evv018 PMC5322547

[bib38] Plough L. V., 2012 Environmental stress increases selection against and dominance of deleterious mutations in inbred families of the Pacific oyster, *Crassostrea gigas*. Mol. Ecol. 21: 3974–3987. 22747636 10.1111/j.1365-294X.2012.05688.x

[bib39] Plough L. V. Hedgecock D., 2011 Quantitative trait locus analysis of stage-specific inbreeding depression in the Pacific oyster *Crassostrea gigas*. Genetics 189: 1473–1486. 21940682 10.1534/genetics.111.131854 PMC3241428

[bib40] Sauvage C. Boudry P. De Koning D.-J. Haley C. S. Heurtebise S., 2010 QTL for resistance to summer mortality and OsHV-1 load in the Pacific oyster (*Crassostrea gigas*). Anim. Genet. 41: 390–399. 20096029 10.1111/j.1365-2052.2009.02018.x

[bib41] Shah R. Cavanagh C. R. Huang B. E., 2014 Computationally efficient map construction in the presence of segregation distortion. Theor. Appl. Genet. 127: 2585–2597. 25260690 10.1007/s00122-014-2401-0

[bib42] Shen R. Fan J. B. Campbell D. Chang W. Chen J., 2005 High-throughput SNP genotyping on universal bead arrays. Mutat. Res. 573: 70–82. 15829238 10.1016/j.mrfmmm.2004.07.022

[bib43] Stam P., 1993 Construction of integrated genetic linkage maps by means of a new computer package: JoinMap. Plant J. 3: 739–744.

[bib44] Truong S. K. McCormick R. F. Morishige D. T. Mullet J. E., 2014 Resolution of genetic map expansion caused by excess heterozygosity in plant recombinant inbred populations. G3 (Bethesda) 4: 1963–1969. 25128435 10.1534/g3.114.012468 PMC4199702

[bib45] Van Ooijen J. W., 2006 *JoinMap 4*, *Software for the calculation of genetic linkage maps in experimental populations*. Kyazma B. V., The Netherlands.

[bib46] Wang C. M. Zhu C. S. Zhai H. Q. Wan J. M., 2005 Mapping segregation distortion loci and quantitative trait loci for spikelet sterility in rice (*Oryza sativa* L.). Genet. Res. 86: 97–106. 16356283 10.1017/S0016672305007779

[bib47] Wang H. Zhang G. Lio X. Guo X. M., 2008 Classification of common oysters from North China. J. Shellfish Res. 27: 495–503.

[bib48] Wu Y. H. Bhat P. R. Close T. J. Lonardi S., 2008 Efficient and accurate construction of genetic linkage maps from the minimum spanning tree of a graph. PLoS Genet. 4: e1000212. 18846212 10.1371/journal.pgen.1000212 PMC2556103

[bib49] Wu Y. H. Close T. J. Lonardi S., 2011 Accurate construction of consensus genetic maps via integer linear programming. IEEE-ACM Trans. Comp. Biol. Bioinform. 8: 381–394. 10.1109/TCBB.2010.35 20479505

[bib50] Zhang G. Fang X. Guo X. Li L. Luo R., 2012 The oyster genome reveals stress adaptation and complexity of shell formation. Nature 490: 49–54. 22992520 10.1038/nature11413

[bib51] Zhong X. X. Li Q. Guo X. Yu H. Kong L., 2014 QTL mapping for glycogen content and shell pigmentation in the Pacific oyster *Crassostrea gigas* using microsatellites and SNPs. Aquacult. Int. 22: 1877–1899.

[bib52] Zhu C. Zhang Y.-M., 2007 An EM algorithm for mapping segregation distortion loci. BMC Genet. 8: 82. 18047652 10.1186/1471-2156-8-82 PMC2257974

[bib53] Zhu C. Wang C. Zhang Y.-M., 2007 Modeling segregation distortion for viability selection I. reconstruction of linkage maps with distorted markers. Theor. Appl. Genet. 114: 295–305. 17119913 10.1007/s00122-006-0432-x

