# Supplementary material for: Second-Generation Linkage Maps for the Pacific Oyster Crassostrea gigas Reveal Errors in Assembly of Genome Scaffolds
Source: G3 (Bethesda). 2015 Aug 6;5(10):2007–19. doi: 10.1534/g3.115.019570 (PMC4592983; doi:10.1534/g3.115.019570)
Supplement: Supporting Information [file supp_5_10_2007__index.html]

Second-Generation Linkage Maps for the Pacific Oyster Crassostrea gigas Reveal Errors in Assembly of Genome Scaffolds — Supporting Information 

# Second-Generation Linkage Maps for the Pacific Oyster *Crassostrea gigas* Reveal Errors in Assembly of Genome Scaffolds

## Supporting Information for Hedgecock *et al.*, 2015

**Files in this Data Supplement:**

- Supporting Information - Figures S1-S2 and Tables S1-S7 (PDF, 179 KB)
- Table S1 - Genotypes at 619 markers in 46 individuals of Pacific oyster G0 family *F12*. (.xlsx, 114 KB)
- Table S2 - Genotypes at 683 markers in 46 individuals of Pacific oyster G0 family *F45*. (.xlsx, 126 KB)
- Table S3 - Genotypes at 636 markers in 46 individuals of Pacific oyster G0 family *F20*. (.xlsx, 118 KB)
- Table S4 - Genotypes at 533 markers in 90 individuals of Pacific oyster F2 family 2×10. (.xlsx, 176 KB)
- Table S5 - Genotypes at 547 markers in 108 individuals of Pacific oyster F2 family 51×35. (.xlsx, 213 KB)
- Table S6 - Mapping of scaffolds and SNPs to linkage groups in five families of Pacific oyster. (.xlsx, 56 KB)
- Table S7 - Consensus linkage map of 656 markers for the Pacific oyster. (.xlsx, 29 KB)
