# Supplementary material for: Second-Generation Linkage Maps for the Pacific Oyster Crassostrea gigas Reveal Errors in Assembly of Genome Scaffolds
Source: G3 (Bethesda). 2015 Aug 6;5(10):2007–19. doi: 10.1534/g3.115.019570 (PMC4592983; doi:10.1534/g3.115.019570)
Supplement: Supporting Information [file supp_g3.115.019570_019570SI.pdf]

G3 · Genes | Genomes | Genetics

Second-Generation Linkage Maps for the Pacific Oyster *Crassostrea gigas* Reveal Errors in Assembly of Genome Scaffolds

Dennis Hedgecock[*], Grace Shin[*], Andrew Y. Gracey[*], David Van Den Berg[§], and Manoj P. Samanta[†]

[*]Department of Biological Sciences, University of Southern California, Los Angeles, CA 90089-0371; [§]Preventive Medicine, Keck School of Medicine, University of Southern California, Los Angeles 90089-9601; [†]Systemix Institute, Redmond, WA 98053 USA

Corresponding author:
Dennis Hedgecock
Department of Biological Sciences
University of Southern California
3616 Trousdale Pkwy
Los Angeles, CA 90089-0371
1 (213) 821-2091
dhedge@usc.edu

DOI: 10.1534/g3.115.019570

Two ML maps illustrate factors influencing marker orders and map lengths

The plausibility of marker positions for two maximum likelihood maps, for all markers and for bi-parentally segregating framework markers, are presented (Figs. S1, S2), in order to illustrate factors influencing map lengths and marker orders. In the first example, LG 1 for family *51×35*, 68 grouped markers are reduced to 66 by removal of two, poorly fitting markers (Table 6). Segregation ratios for the remaining 66 markers conform to Mendelian expectations (bar charts on right margins of main tables showing plausibility of marker positions; only one marker on the maternal linkage map is significant at the $P < 0.05$ level), so selection is not a factor in this map construction. Still, some moderately large nearest-neighbor fits (5-10 cM) are evident over the first half of each parental ML map, mostly associated with single-parent markers (bar charts along bottom margin of main tables in Figs. S1A, S1B). In addition, single-parent markers at the termini of both parental maps expand lengths from about 70 cM to over 200 cM. On the other hand, framework RG and ML maps, constructed with 32 markers, are similar in length (58.7 cM *v*. 62.8 cM, Table 7; Fig. S1C).

In the second example (79 markers grouped for LG 10 of family *2×10*), clusters of markers with severely distorted Mendelian ratios are associated with large jumps in length and regions of very poor fit on the two ML parental maps (Figs. S2A, S2B). Note, first, the important changes of scale, from Fig. S1, in both the right-margin bar charts, showing agreement with Mendelian ratios, and the bottom-margin bar charts, showing nearest neighbor fit (on a $\log_{10}$ scale). Note, also, however, the association of single parent markers with regions of poor fit and length expansion. For this example, restricting maps to 36 framework markers does not solve the problem of excessive map length and poor nearest-neighbor fit (not shown). Removal of an additional 12 framework markers with highly distorted segregation ratios is required to achieve

congruence of marker orders for RG and ML framework maps (Fig. 5). Still, this reduced ML framework map has one region of poor fit (Fig. S2C) and is 2.4× as long as the comparable RG map (Table 7).

FIGURE LEGENDS

Figure S1. Plausibility of marker positions for linkage group 1 of F_2 family *51×35* (proportions of 1000 simulations of marker positions in body of tables). Bar charts on right margins of main tables show probability ($-\log_{10}P$, from 0 to 1.0 or 1.5) of chi-square goodness-of-fit test for Mendelian proportions at each locus. Bar chart hanging from bottom margins of main tables show nearest-neighbor fit (from 0 cM to 10 cM). (A) Markers (*n*=47) segregating from the male parent; (B) markers (*n*=52) segregating from the female parent; (C) framework markers (*n*=32) segregating from both parents.

Figure S2. Plausibility of marker positions for linkage group 10 of family *2×10* (proportions of 1000 simulations of marker positions in body of tables). Bar charts on right margins of main tables show probability ($-\log_{10}P$, from 0 to 15) of chi-square goodness-of-fit test for Mendelian proportions at each locus. Bar chart hanging from bottom margins of main tables give $\log_{10}$ of nearest-neighbor fit (from 0 to 4 [10^4 cM]. (A) Markers (*n*=56) segregating from the male parent; (B) markers (*n*=59) segregating from the female parent; (C) framework markers (*n*=24) segregating from both parents.

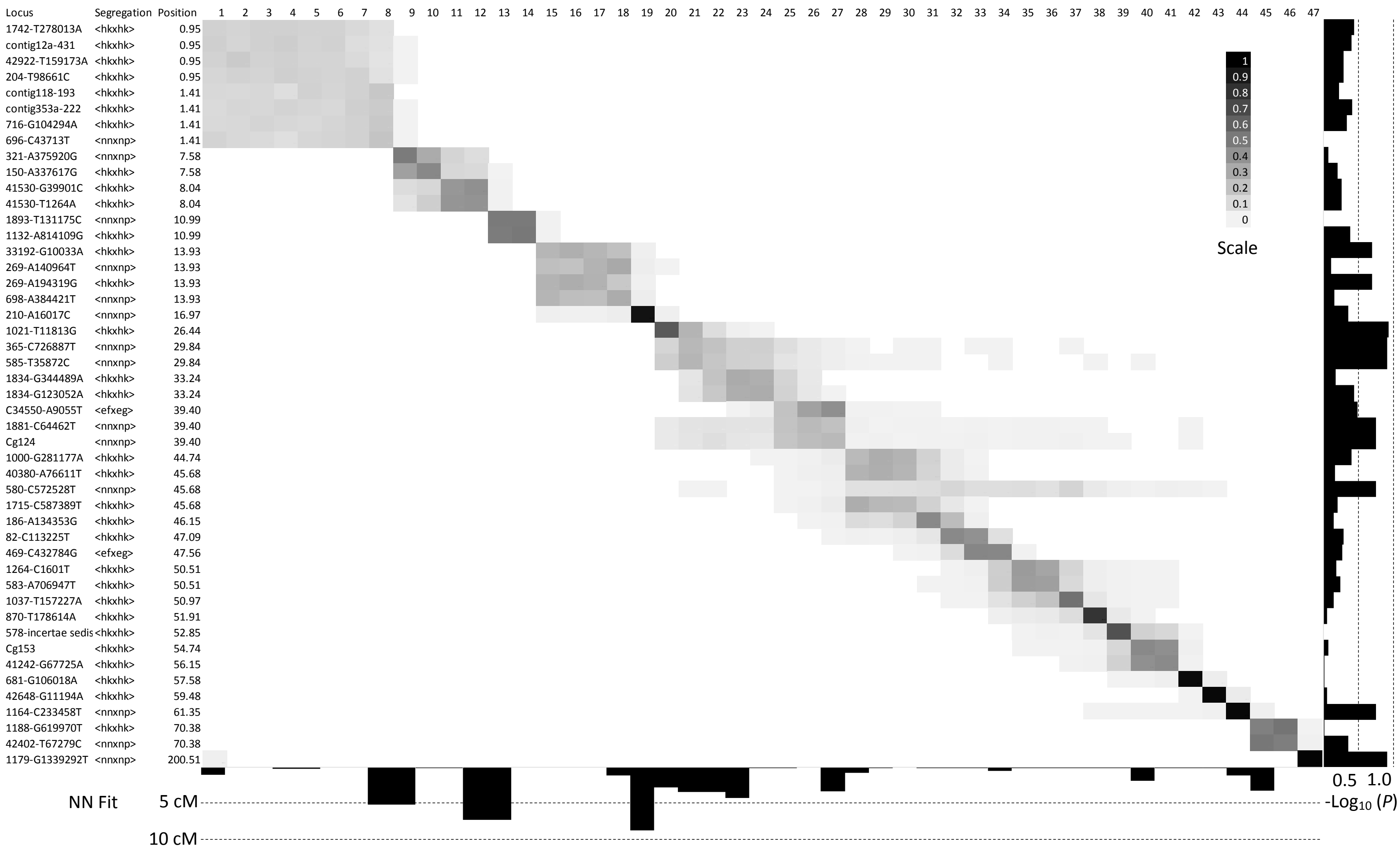
Locus
Segregation
Position
Scale
NN Fit
5 cM
10 cM
0.5 1.0
-Log10 (P)

Figure S1A

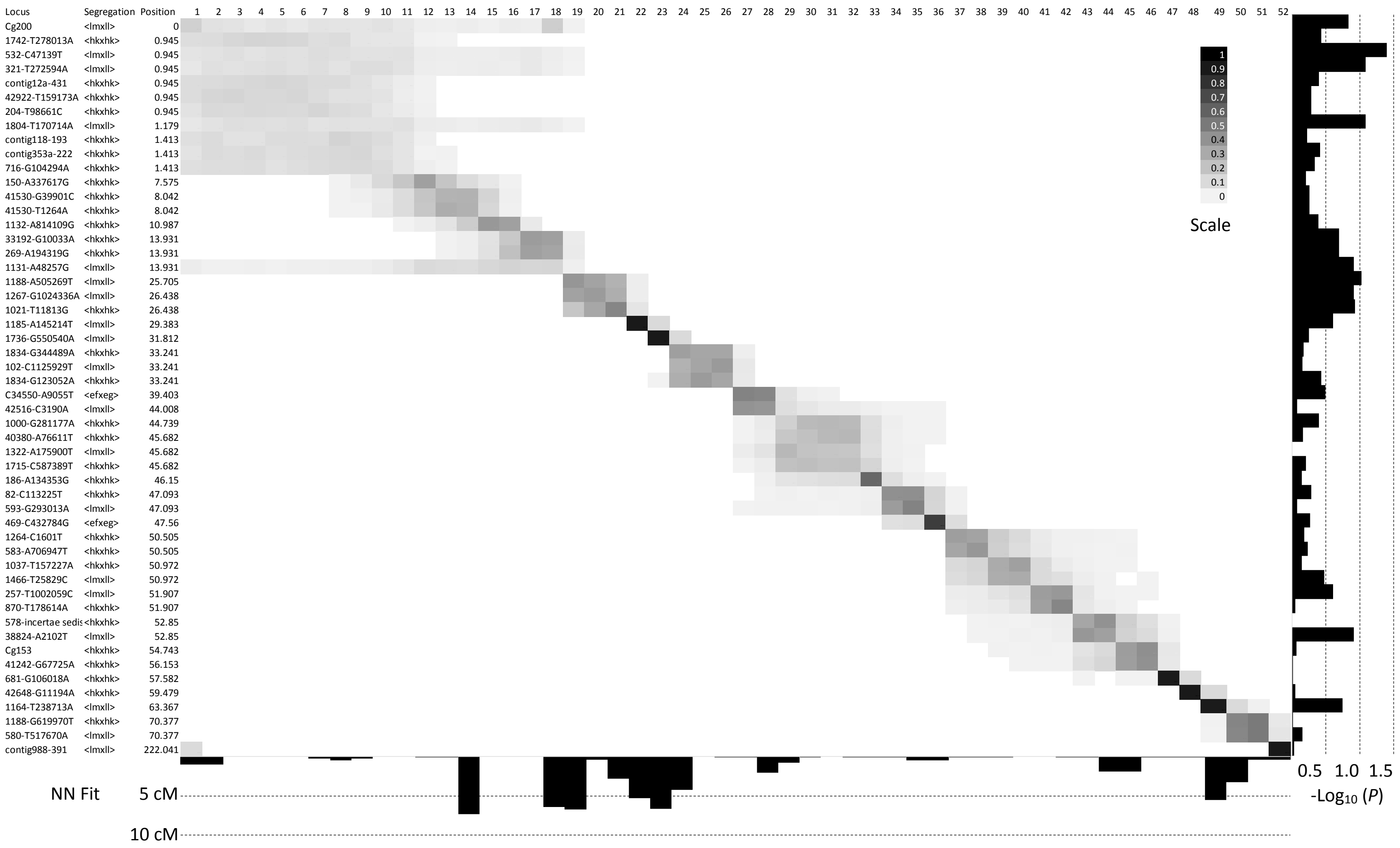
Locus Segregation Position
1 2 3 4 5 6 7 8 9 10 11 12 13 14 15 16 17 18 19 20 21 22 23 24 25 26 27 28 29 30 31 32 33 34 35 36 37 38 39 40 41 42 43 44 45 46 47 48 49 50 51 52
Cg200 <lmxll> 0
1742-T278013A <hkxhk> 0.945
532-C47139T <lmxll> 0.945
321-T272594A <lmxll> 0.945
contig12a-431 <hkxhk> 0.945
42922-T159173A <hkxhk> 0.945
204-T98661C <hkxhk> 0.945
1804-T170714A <lmxll> 1.179
contig118-193 <hkxhk> 1.413
contig353a-222 <hkxhk> 1.413
716-G104294A <hkxhk> 1.413
150-A337617G <hkxhk> 7.575
41530-G39901C <hkxhk> 8.042
41530-T1264A <hkxhk> 8.042
1132-A814109G <hkxhk> 10.987
33192-G10033A <hkxhk> 13.931
269-A194319G <hkxhk> 13.931
1131-A48257G <lmxll> 13.931
1188-A505269T <lmxll> 25.705
1267-G1024336A <lmxll> 26.438
1021-T11813G <hkxhk> 26.438
1185-A145214T <lmxll> 29.383
1736-G550540A <lmxll> 31.812
1834-G344489A <hkxhk> 33.241
102-C1125929T <lmxll> 33.241
1834-G123052A <hkxhk> 33.241
C34550-A9055T <efxeg> 39.403
42516-C3190A <lmxll> 44.008
1000-G281177A <hkxhk> 44.739
40380-A76611T <hkxhk> 45.682
1322-A175900T <lmxll> 45.682
1715-C587389T <hkxhk> 45.682
186-A134353G <hkxhk> 46.15
82-C113225T <hkxhk> 47.093
593-G293013A <lmxll> 47.093
469-C432784G <efxeg> 47.56
1264-C1601T <hkxhk> 50.505
583-A706947T <hkxhk> 50.505
1037-T157227A <hkxhk> 50.972
1466-T25829C <lmxll> 50.972
257-T1002059C <lmxll> 51.907
870-T178614A <hkxhk> 51.907
578-incertae sedis <hkxhk> 52.85
38824-A2102T <lmxll> 52.85
Cg153 <hkxhk> 54.743
41242-G67725A <hkxhk> 56.153
681-G106018A <hkxhk> 57.582
42648-G11194A <hkxhk> 59.479
1164-T238713A <lmxll> 63.367
1188-G619970T <hkxhk> 70.377
580-T517670A <lmxll> 70.377
contig988-391 <lmxll> 222.041
1
0.9
0.8
0.7
0.6
0.5
0.4
0.3
0.2
0.1
0
Scale
NN Fit
5 cM
10 cM
0.5 1.0 1.5
-Log10 (P)

Figure S1B

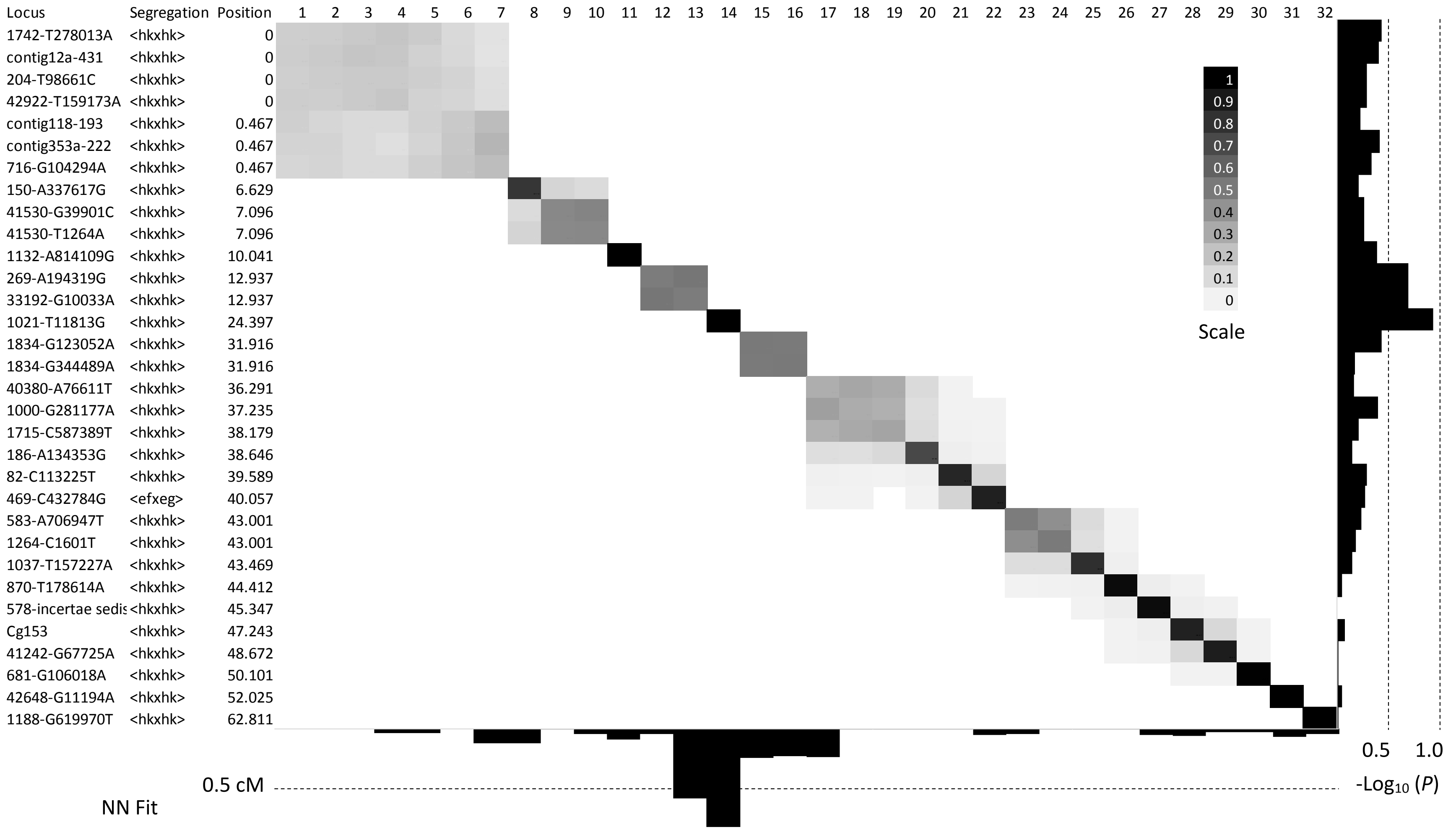
Locus
Segregation
Position
1 2 3 4 5 6 7 8 9 10 11 12 13 14 15 16 17 18 19 20 21 22 23 24 25 26 27 28 29 30 31 32
1742-T278013A <hkxhk> 0
contig12a-431 <hkxhk> 0
204-T98661C <hkxhk> 0
42922-T159173A <hkxhk> 0
contig118-193 <hkxhk> 0.467
contig353a-222 <hkxhk> 0.467
716-G104294A <hkxhk> 0.467
150-A337617G <hkxhk> 6.629
41530-G39901C <hkxhk> 7.096
41530-T1264A <hkxhk> 7.096
1132-A814109G <hkxhk> 10.041
269-A194319G <hkxhk> 12.937
33192-G10033A <hkxhk> 12.937
1021-T11813G <hkxhk> 24.397
1834-G123052A <hkxhk> 31.916
1834-G344489A <hkxhk> 31.916
40380-A76611T <hkxhk> 36.291
1000-G281177A <hkxhk> 37.235
1715-C587389T <hkxhk> 38.179
186-A134353G <hkxhk> 38.646
82-C113225T <hkxhk> 39.589
469-C432784G <efxeg> 40.057
583-A706947T <hkxhk> 43.001
1264-C1601T <hkxhk> 43.001
1037-T157227A <hkxhk> 43.469
870-T178614A <hkxhk> 44.412
578-incertae sedis <hkxhk> 45.347
Cg153 <hkxhk> 47.243
41242-G67725A <hkxhk> 48.672
681-G106018A <hkxhk> 50.101
42648-G11194A <hkxhk> 52.025
1188-G619970T <hkxhk> 62.811
1
0.9
0.8
0.7
0.6
0.5
0.4
0.3
0.2
0.1
0
Scale
0.5 1.0
-Log10 (P)
0.5 cM
NN Fit
1.0 cM

Figure S1C

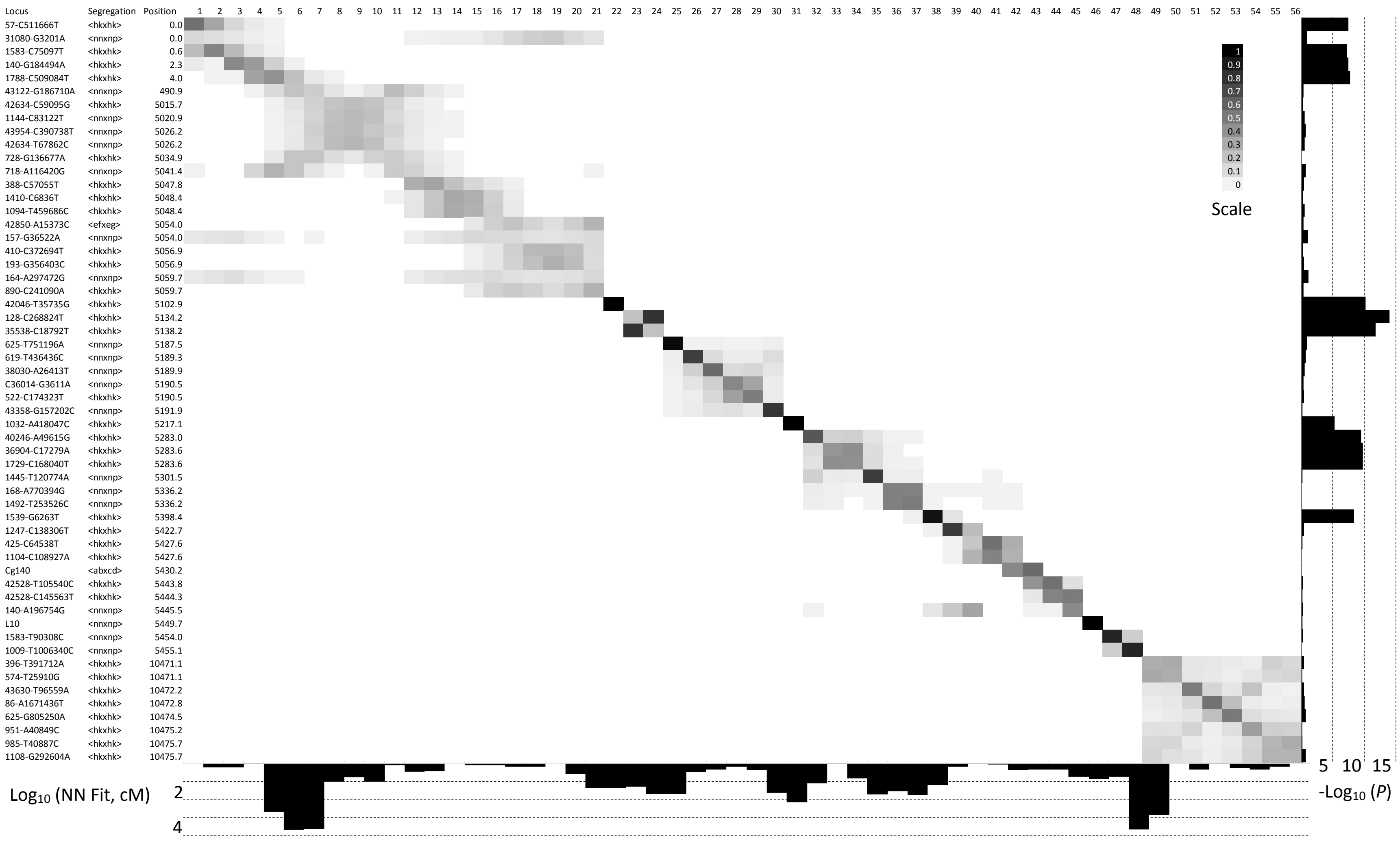

Locus Segregation Position
57-C511666T <hkxhk> 0.0
31080-G3201A <nnxnp> 0.0
1583-C75097T <hkxhk> 0.6
140-G184494A <hkxhk> 2.3
1788-C509084T <hkxhk> 4.0
43122-G186710A <nnxnp> 490.9
42634-C59095G <hkxhk> 5015.7
1144-C83122T <nnxnp> 5020.9
43954-C390738T <nnxnp> 5026.2
42634-T67862C <nnxnp> 5026.2
728-G136677A <hkxhk> 5034.9
718-A116420G <nnxnp> 5041.4
388-C57055T <hkxhk> 5047.8
1410-C6836T <hkxhk> 5048.4
1094-T459686C <hkxhk> 5048.4
42850-A15373C <efxeg> 5054.0
157-G36522A <nnxnp> 5054.0
410-C372694T <hkxhk> 5056.9
193-G356403C <hkxhk> 5056.9
164-A297472G <nnxnp> 5059.7
890-C241090A <hkxhk> 5059.7
42046-T35735G <hkxhk> 5102.9
128-C268824T <hkxhk> 5134.2
35538-C18792T <hkxhk> 5138.2
625-T751196A <nnxnp> 5187.5
619-T436436C <nnxnp> 5189.3
38030-A26413T <nnxnp> 5189.9
C36014-G3611A <nnxnp> 5190.5
522-C174323T <hkxhk> 5190.5
43358-G157202C <nnxnp> 5191.9
1032-A418047C <hkxhk> 5217.1
40246-A49615G <hkxhk> 5283.0
36904-C17279A <hkxhk> 5283.6
1729-C168040T <hkxhk> 5283.6
1445-T120774A <nnxnp> 5301.5
168-A770394G <nnxnp> 5336.2
1492-T253526C <nnxnp> 5336.2
1539-G6263T <hkxhk> 5398.4
1247-C138306T <hkxhk> 5422.7
425-C64538T <hkxhk> 5427.6
1104-C108927A <hkxhk> 5427.6
Cg140 <abxcd> 5430.2
42528-T105540C <hkxhk> 5443.8
42528-C145563T <hkxhk> 5444.3
140-A196754G <nnxnp> 5445.5
L10 <nnxnp> 5449.7
1583-T90308C <nnxnp> 5454.0
1009-T1006340C <nnxnp> 5455.1
396-T391712A <hkxhk> 10471.1
574-T25910G <hkxhk> 10471.1
43630-T96559A <hkxhk> 10472.2
86-A1671436T <hkxhk> 10472.8
625-G805250A <hkxhk> 10474.5
951-A40849C <hkxhk> 10475.2
985-T40887C <hkxhk> 10475.7
1108-G292604A <hkxhk> 10475.7
1 2 3 4 5 6 7 8 9 10 11 12 13 14 15 16 17 18 19 20 21 22 23 24 25 26 27 28 29 30 31 32 33 34 35 36 37 38 39 40 41 42 43 44 45 46 47 48 49 50 51 52 53 54 55 56
1 0.9 0.8 0.7 0.6 0.5 0.4 0.3 0.2 0.1 0
Scale
5 10 15
-Log10 (P)
Log10 (NN Fit, cM)
2
4

Figure S2A

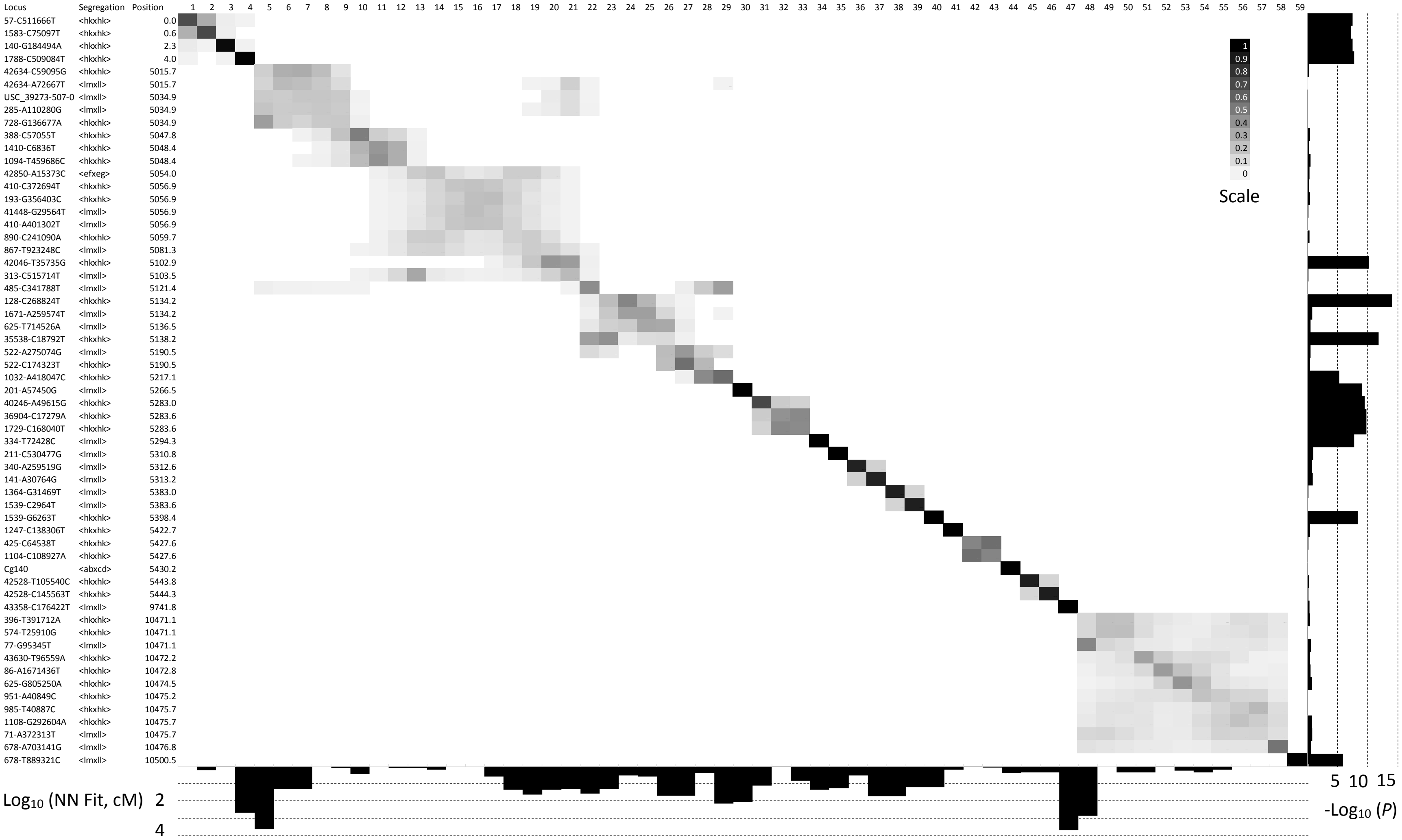
Locus Segregation Position
1 2 3 4 5 6 7 8 9 10 11 12 13 14 15 16 17 18 19 20 21 22 23 24 25 26 27 28 29 30 31 32 33 34 35 36 37 38 39 40 41 42 43 44 45 46 47 48 49 50 51 52 53 54 55 56 57 58 59
57-C511666T <hkxhk> 0.0
1583-C75097T <hkxhk> 0.6
140-G184494A <hkxhk> 2.3
1788-C509084T <hkxhk> 4.0
42634-C59095G <hkxhk> 5015.7
42634-A72667T <lmxll> 5015.7
USC_39273-507-0 <lmxll> 5034.9
285-A110280G <lmxll> 5034.9
728-G136677A <hkxhk> 5034.9
388-C57055T <hkxhk> 5047.8
1410-C6836T <hkxhk> 5048.4
1094-T459686C <hkxhk> 5048.4
42850-A15373C <efxeg> 5054.0
410-C372694T <hkxhk> 5056.9
193-G356403C <hkxhk> 5056.9
41448-G29564T <lmxll> 5056.9
410-A401302T <lmxll> 5056.9
890-C241090A <hkxhk> 5059.7
867-T923248C <lmxll> 5081.3
42046-T35735G <hkxhk> 5102.9
313-C515714T <lmxll> 5103.5
485-C341788T <lmxll> 5121.4
128-C268824T <hkxhk> 5134.2
1671-A259574T <lmxll> 5134.2
625-T714526A <lmxll> 5136.5
35538-C18792T <hkxhk> 5138.2
522-A275074G <lmxll> 5190.5
522-C174323T <hkxhk> 5190.5
1032-A418047C <hkxhk> 5217.1
201-A57450G <lmxll> 5266.5
40246-A49615G <hkxhk> 5283.0
36904-C17279A <hkxhk> 5283.6
1729-C168040T <hkxhk> 5283.6
334-T72428C <lmxll> 5294.3
211-C530477G <lmxll> 5310.8
340-A259519G <lmxll> 5312.6
141-A30764G <lmxll> 5313.2
1364-G31469T <lmxll> 5383.0
1539-C2964T <lmxll> 5383.6
1539-G6263T <hkxhk> 5398.4
1247-C138306T <hkxhk> 5422.7
425-C64538T <hkxhk> 5427.6
1104-C108927A <hkxhk> 5427.6
Cg140 <abxcd> 5430.2
42528-T105540C <hkxhk> 5443.8
42528-C145563T <hkxhk> 5444.3
43358-C176422T <lmxll> 9741.8
396-T391712A <hkxhk> 10471.1
574-T25910G <hkxhk> 10471.1
77-G95345T <lmxll> 10471.1
43630-T96559A <hkxhk> 10472.2
86-A1671436T <hkxhk> 10472.8
625-G805250A <hkxhk> 10474.5
951-A40849C <hkxhk> 10475.2
985-T40887C <hkxhk> 10475.7
1108-G292604A <hkxhk> 10475.7
71-A372313T <lmxll> 10475.7
678-A703141G <lmxll> 10476.8
678-T889321C <lmxll> 10500.5
1 0.9 0.8 0.7 0.6 0.5 0.4 0.3 0.2 0.1 0
Scale
5 10 15
-Log10 (P)
Log10 (NN Fit, cM) 2 4

Figure S2B

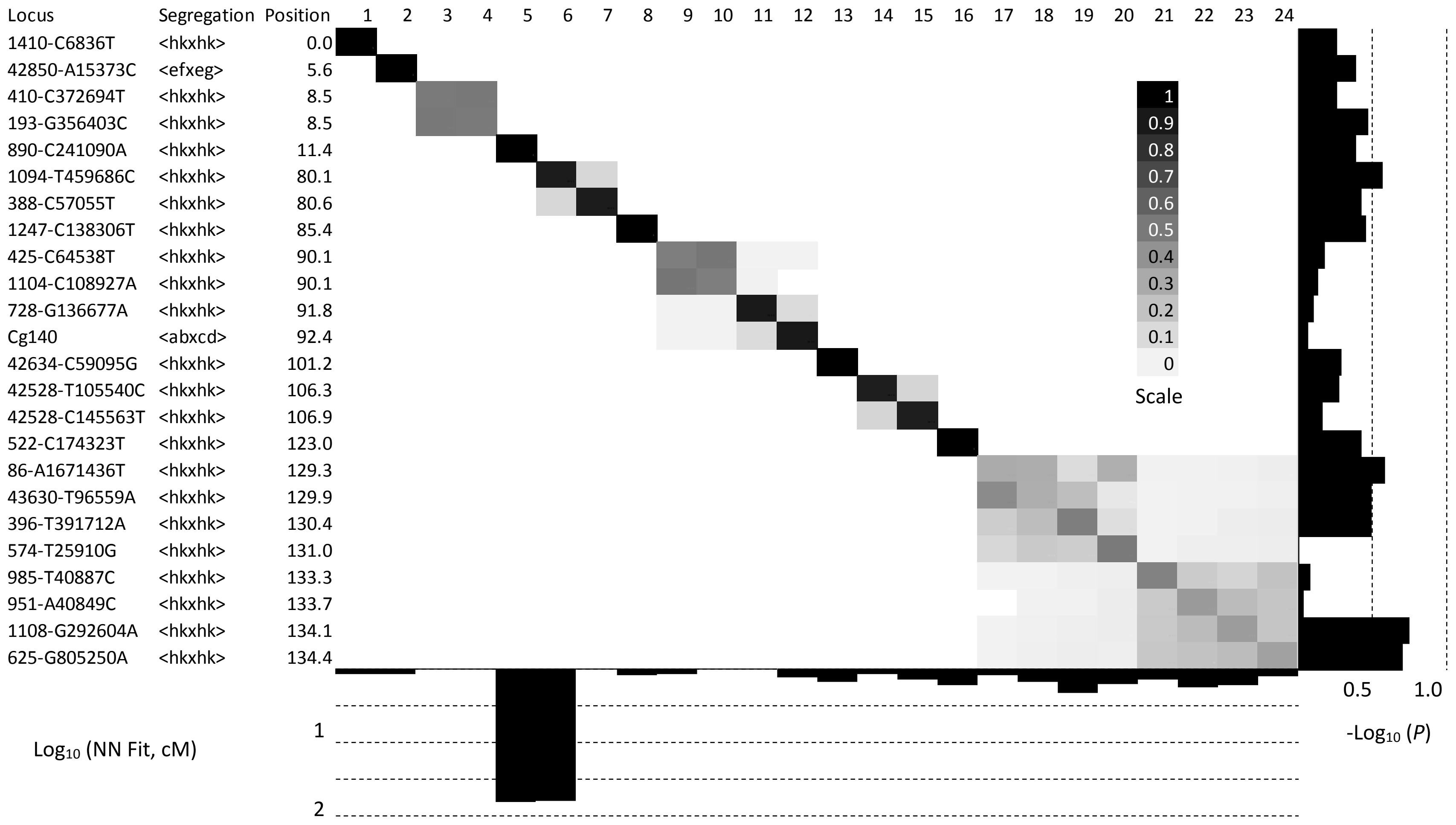
Locus
Segregation
Position
1 2 3 4 5 6 7 8 9 10 11 12 13 14 15 16 17 18 19 20 21 22 23 24
1410-C6836T <hkxhk> 0.0
42850-A15373C <efxeg> 5.6
410-C372694T <hkxhk> 8.5
193-G356403C <hkxhk> 8.5
890-C241090A <hkxhk> 11.4
1094-T459686C <hkxhk> 80.1
388-C57055T <hkxhk> 80.6
1247-C138306T <hkxhk> 85.4
425-C64538T <hkxhk> 90.1
1104-C108927A <hkxhk> 90.1
728-G136677A <hkxhk> 91.8
Cg140 <abxcd> 92.4
42634-C59095G <hkxhk> 101.2
42528-T105540C <hkxhk> 106.3
42528-C145563T <hkxhk> 106.9
522-C174323T <hkxhk> 123.0
86-A1671436T <hkxhk> 129.3
43630-T96559A <hkxhk> 129.9
396-T391712A <hkxhk> 130.4
574-T25910G <hkxhk> 131.0
985-T40887C <hkxhk> 133.3
951-A40849C <hkxhk> 133.7
1108-G292604A <hkxhk> 134.1
625-G805250A <hkxhk> 134.4
1
0.9
0.8
0.7
0.6
0.5
0.4
0.3
0.2
0.1
0
Scale
0.5 1.0
-Log10 (P)
Log10 (NN Fit, cM)
1
2

Figure S2C

Supplementary Tables

Tables S1-S7 are compiled in an Excel spreadsheet, available for download at be www.g3journal.org/lookup/suppl/doi:10.1534/g3.115.019570/-/DC1. Tables S1-S5 are the genotype data, which were entered into JoinMap 4, for each of the five mapping families; not all markers necessarily ended up on maps. JoinMap codes *lm*x*ll* and *nn*x*np* actually correspond to male and female parents, respectively, and are reported as *np*x*nn* and *ll*x*lm* in Table 1 of main text.

Table S1. Genotypes at 619 markers in 46 individuals of Pacific oyster G_0 family F12.

Table S2. Genotypes at 683 markers in 46 individuals of Pacific oyster G_0 family F45.

Table S3. Genotypes at 636 markers in 46 individuals of Pacific oyster G_0 family F20.

Table S4. Genotypes at 533 markers in 90 individuals of Pacific oyster F_2 family 2×10.

Table S5. Genotypes at 547 markers in 108 individuals of Pacific oyster F_2 family 51×35.

Table S6. Mapping of scaffolds and SNPs to linkage groups in five families of Pacific oyster.

Table S7. Consensus linkage map of 656 markers for the Pacific oyster.
